# Geologic Constraints on the Formation and Evolution of Saturn’s Mid-Sized Moons

**DOI:** 10.1007/s11214-024-01084-z

**Published:** 2024-07-17

**Authors:** Alyssa Rose Rhoden, Sierra N. Ferguson, William Bottke, Julie C. Castillo-Rogez, Emily Martin, Michael Bland, Michelle Kirchoff, Marco Zannoni, Nicolas Rambaux, Julien Salmon

**Affiliations:** 1 Southwest Research Institute, 1050 Walnut St, Boulder, CO 80302 USA; 2 Jet Propulsion Laboratory, California Institute of Technology, Pasadena, CA USA; 3 Center for Earth and Planetary Studies, National Air and Space Museum, Smithsonian Institution, Washington, DC USA; 4 U.S. Geological Survey, Astrogeology Science Center, Flagstaff, AZ USA; 5 Dipartimento di Ingegneria Industriale, Alma Mater Studiorum – Università di Bologna, Forlì, Italy; 6 IMCCE, CNRS, Observatoire de Paris, PSL Université, Sorbonne Université, Université de Lille 1, UMR 8028 du CNRS, 77 Denfert-Rochereau, 75014 Paris, France

**Keywords:** Saturnian satellites, Icy moons, Geology, Tectonics, Cratering, Interiors, Ocean worlds, Mimas, Enceladus, Tethys, Dione, Rhea

## Abstract

Saturn’s mid-sized icy moons have complex relationships with Saturn’s interior, the rings, and with each other, which can be expressed in their shapes, interiors, and geology. Observations of their physical states can, thus, provide important constraints on the ages and formation mechanism(s) of the moons, which in turn informs our understanding of the formation and evolution of Saturn and its rings. Here, we describe the cratering records of the mid-sized moons and the value and limitations of their use for constraining the histories of the moons. We also discuss observational constraints on the interior structures of the moons and geologically-derived inferences on their thermal budgets through time. Overall, the geologic records of the moons (with the exception of Mimas) include evidence of epochs of high heat flows, short- and long-lived subsurface oceans, extensional tectonics, and considerable cratering. Curiously, Mimas presents no clear evidence of an ocean within its surface geology, but its rotation and orbit indicate a present-day ocean. While the moons need not be primordial to produce the observed levels of interior evolution and geologic activity, there is likely a minimum age associated with their development that has yet to be determined. Uncertainties in the populations impacting the moons makes it challenging to further constrain their formation timeframes using craters, whereas the characteristics of their cores and other geologic inferences of their thermal evolutions may help narrow down their potential histories. Disruptive collisions may have also played an important role in the formation and evolution of Saturn’s mid-sized moons, and even the rings of Saturn, although more sophisticated modeling is needed to determine the collision conditions that produce rings and moons that fit the observational constraints. Overall, the existence and physical characteristics of Saturn’s mid-sized moons provide critical benchmarks for the development of formation theories.

## Introduction

**Fig. 1:**
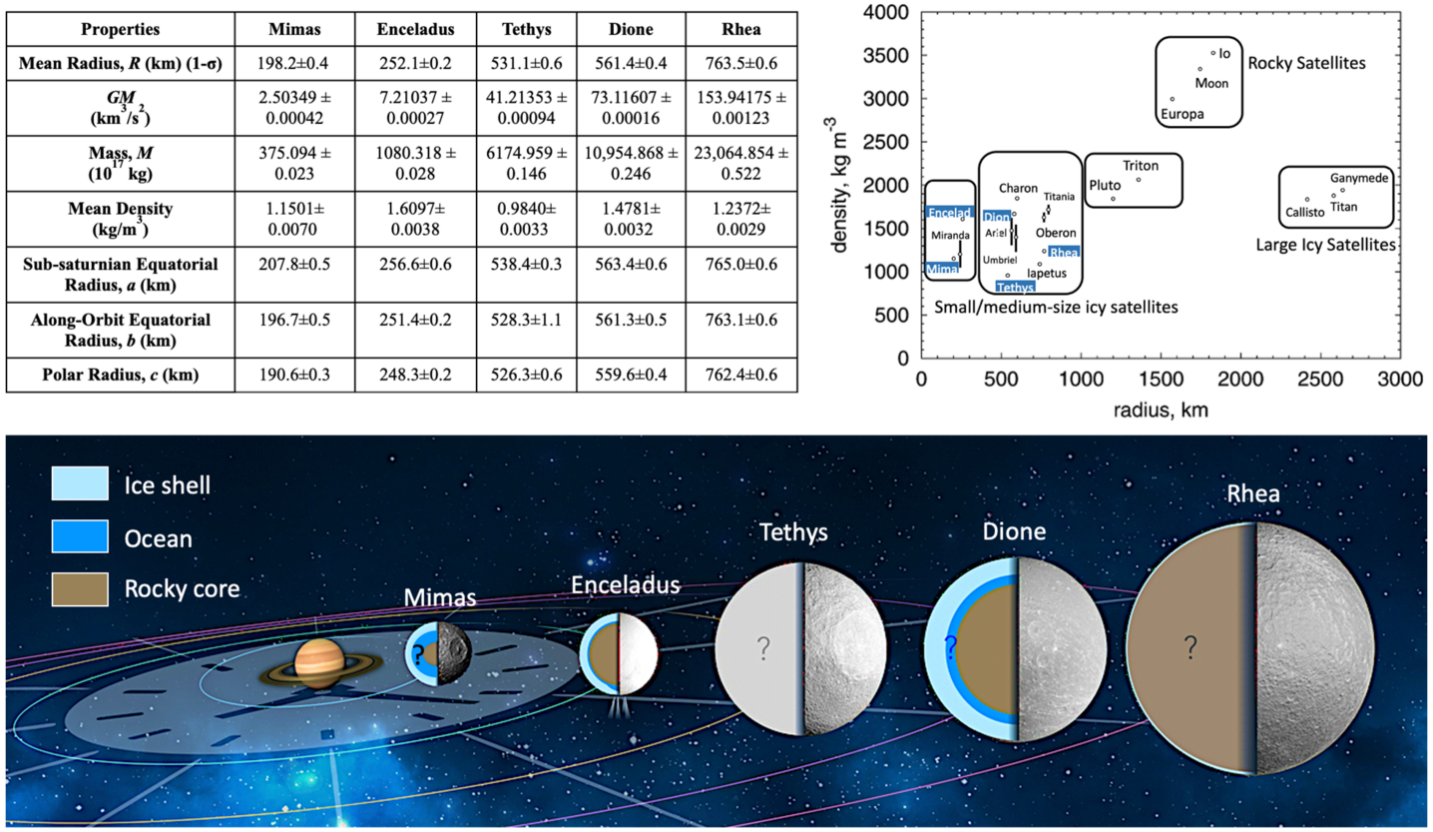
(Top left) Key physical properties of Mimas, Enceladus, Tethys, Dione, and Rhea (error bars are 1-sigma); (Top right) comparison of their densities and mean radii to those of other moons in the outer solar system. References for radii and densities are Thomas (2010) and Archinal et al. (2018). (Bottom) Summary of our knowledge of their internal structures. There are still major uncertainties about the state of differentiation of Rhea and Tethys. Modified from Neveu and Rhoden (2019)

Saturn possesses a diverse system of satellites, including moonlets embedded within the rings, a suite of mid-sized icy moons (Mimas, Enceladus, Tethys, Dione, and Rhea), one large moon that possesses a thick atmosphere and a global hydrological cycle (Titan), distant moons with puzzling properties (Hyperion, Iapetus, Phoebe), and several populations of distant irregular satellites. In addition, two of the mid-sized moons share their orbits with co-orbital moons (Calypso/Telesto at Tethys and Helene/Polydeuces at Dione). The *Cassini-Huygens* mission, building upon the data sets acquired by *Voyager*, revealed many of the physical, dynamical, geological, and compositional properties of these moons (Fig. 1; Table 1), although many questions regarding their origins, interiors, and habitability still remain.

**Table 1:** Key orbital properties of Saturn’s large moons (Source: ssd.jpl.nasa.gov)

Properties	Mimas	Enceladus	Tethys	Dione	Rhea
Semi-Major Axis, *a* (km)	186,000	238,400	295,000	377,700	527,200
Orbital Eccentricity, *e*	0.020	0.005	0.0001	0.002	0.001
Orbital Inclination, *i* (°)	1.6	0.0	1.1	0.0	0.3
Orbital Period (days)	0.942422	1.370218	1.887802	2.736916	4.517503

A peculiarity of Saturn’s mid-sized moons is that they do not display a density gradient with distance from the planet (Fig. 1), unlike the regular moons of Jupiter. The moon with the highest density is Enceladus, which orbits between the two lowest density moons: Mimas (interior) and Tethys (exterior). Enceladus’ density corresponds to a rock mass fraction of 60%, assuming a dry rock composition akin to a CI chondrite (Castillo-Rogez et al. 2018). In contrast, Tethys’ rock fraction is limited to only ∼7% by mass, although it could be as much as 15–20% if Tethys maintains substantial porosity (e.g., Thomas 2010; Castillo-Rogez et al. 2018), and Mimas’ density implies only ∼9% rock, by mass. While the densities and inferred rock fractions vary wildly, the moons increase in mass and radius with distance from Saturn (Fig. 1), which may be a clue as to the processes that led Saturn’s moons to display such different characteristics than the moons of Jupiter. In addition, our new understanding of dissipation within Saturn has dramatically altered our interpretations of the system, opening up new evolutionary pathways not previously considered.

Dissipation within Saturn, described by the parameter Q, exerts a strong control on the orbital migration rates of its moons. The “classical” value of Saturn’s Q is ∼18,000 (e.g., Meyer and Wisdom 2008), which was derived assuming that the moons evolved to their current orbital distances over the age of the solar system. However, analyses of a combination of historical astrometry measurements and contemporary *Cassini* data (Lainey et al. 2012, 2017, 2020) have revealed much higher orbital migration rates for the moons, from Mimas to Titan, which imply that Saturn’s Q is much lower than the classical value. In fact, the orbital migration rates of the mid-sized moons each indicate a different tidal Q (Lainey et al. 2017, 2020), ranging from ∼300 to ∼2500 (Lainey et al. 2012; Nimmo et al. 2018; Lainey et al. 2017, 2020). It may be the case that Saturn’s Q changes with time and/or that Saturn’s Q is frequency-dependent, such that the moons experience a different effective tidal Q as their orbits expand (e.g., Fuller et al. 2016; Nimmo et al. 2018; Lainey et al. 2020). This new view of Saturn’s Q allows for the possibility that the mid-sized moons can be much younger than the age of the Solar System and still have evolved to their present-day orbits (Lainey et al. 2020). However, ancient moons are not ruled out by a lower Q of Saturn.

In parallel with these findings, a plethora of new hypotheses have been put forth to explain the formation of the mid-sized moons inward of Titan that do not invoke contemporaneous formation with Saturn from its circumplanetary disk. In particular, it has been suggested that at least one, and possibly all, of these moons were spawned from Saturn’s rings (Canup 2010; Charnoz et al. 2011; Crida and Charnoz 2012; Salmon and Canup 2017; Dubinski 2019). In that case, the age of the rings and the ages of the moons are inextricably linked. A second suite of hypotheses invoke disruptive collisions into or among a previous generation of moons, with the debris coalescing to become the present-day mid-sized moons (e.g., Ćuk et al. 2016; Asphaug and Reufer 2013; Teodoro et al. 2023). We discuss these models in more detail in the following section.

The new vision of Saturn’s Q, along with the breadth of formation models for the mid-sized moons, has resulted in a range of allowable ages for the moons from only ∼100 Myr to nearly the age of Saturn itself. Here, we turn to the geophysical characteristics of Mimas, Enceladus, Tethys, Dione, and Rhea, to obtain additional constraints on both the timing and mechanism of their formation and subsequent histories. This chapter focuses on the mid-sized icy moons found within Titan’s orbit, in part because they are more diagnostic of processes within Saturn and its ring-moon system than more distant moons. These bodies are also particularly intriguing because they may all have had subsurface oceans, some of which persist to the present day, despite their small sizes (radii between ∼200 and 750 km) and relatively rock-poor interiors (Fig. 1); we discuss the evidence for past and contemporary oceans in the following sections. Taken together, Saturn’s mid-sized moons provide an opportunity to explore a wide range of conditions that may promote ocean development and the development of habitable worlds. In addition, the mid-sized moons can inform our understanding of satellite formation and the potential links between dense rings and mid-sized moons, which are also observed at Uranus.

We summarize the present hypotheses for the formation of Saturn’s mid-sized moons (Sect. 2) and then describe the present-day geophysical observations that provide constraints on the histories of the moons (Sects. 3–5). Specifically, we explore the cratering records of the mid-sized moons, as well as their surface geology, interior structures, and thermal budgets. We conclude with a summary intended to guide the development and testing of theories for the moons’ formation and evolution (Sect. 6). Because this review covers a wide breadth of topics, where applicable, we point to reviews already in the literature to provide the reader a starting point from which to delve deeper into these topics. In particular, we refer to the reader to Schenk et al. (2018), Kirchoff et al. (2018), and Patterson et al. (2018) for surface geology of the mid-sized moons and to Castillo-Rogez et al. (2018) and Hemingway et al. (2018) for interior structures of the mid-sized moons; the latter also includes a thorough description of the methodology used to infer interior structure (e.g., through gravity measurements).

## Hypotheses for the Origins of Saturn’s Mid-Sized Moons

Moon formation within a circumplanetary disk (CPD) is a well-established idea (e.g., Canup and Ward 2009; Mosqueira et al. 2010 and references therein). However, Saturn’s mid-sized moons grow in both mass and radius with distance from Saturn, whilst displaying variable densities (Fig. 1), which is not the pattern observed among the regular moons of Jupiter nor the expected outcome of CPD formation (e.g., Canup and Ward 2009; Mosqueira et al. 2010). Hence, as we discuss in this section, many studies have investigated whether the present mid-sized moons (Mimas through Rhea) initially formed by a different mechanism and/or manifested from disruptions into or among precursor moons.

One suite of models posits that the mid-sized moons accreted in Saturn’s rings (Charnoz et al. 2010; Canup 2010; Charnoz et al. 2011; Crida and Charnoz 2012; Salmon and Canup 2017). In that case, one moon forms, grows, and migrates out of the rings before the next. A natural outcome of the model is that the sizes of the newly formed moons would decrease over time, as the remaining mass in the ring decreases, which could explain the observed mass gradient of the moons. In particular, Crida and Charnoz (2012) showed that the relative masses of the moons would naturally result from the merging of similarly-sized moons during formation from the rings. The densities of the moons, which are set by their rock fractions, are determined somewhat stochastically in these models; either rock “seeds” present in the rings become the cores of the moon (e.g., Charnoz et al. 2011) or the rocky material is brought in by impacts over time (Salmon and Canup 2017).

Ring-born moon scenarios require that a massive ring around Saturn predates any mid-sized moons that emerged from it, and imply that the moons are different ages. Some models invoke ring formation for all of the moons (Charnoz et al. 2011; Crida and Charnoz 2012) – with Rhea being as much as a billion years older than Mimas (Charnoz et al. 2011) – while others suggest that only Mimas, Enceladus, and Tethys formed in this manner (Salmon and Canup 2017). In either case, the ages of the mid-sized moons would be tied to the age of the rings, which we will now briefly discuss.

Canup (2010) proposed that Saturn’s rings formed from a Titan-sized body that accreted in the CPD, migrated inward due to interactions with the gas disk, and was disrupted when crossing Saturn’s Roche limit. The study assumed that the moon’s outer ice layers were tidally stripped to form massive icy rings while the bulk of the moon’s rocky core was lost to the planet, resulting in a low rock abundance in the rings. Reliance on gas-driven migration means this process must have occurred early in Saturn’s history. Alternatively Saturn’s rings may have formed from tidal disruption of a large transneptunian object (e.g., Centaur) that crossed Saturn’s Roche’s limit (e.g., Dones 1991; Hyodo and Charnoz 2017). It has not been possible to discriminate between these two scenarios based on the *Cassini* data.

The Canup (2010) model for ring formation, coupled with ring-born moon models (Charnoz et al. 2010; Canup 2010; Charnoz et al. 2011; Crida and Charnoz 2012; Salmon and Canup 2017), implies that the mid-sized moon formation began within ∼1 Gyr after the dissipation of the gas disk around Saturn, roughly 4.5 Gyr ago (see Canup 2010, *Supplementary Information*). Invoking the disruption of an interloper (e.g., Dones 1991; Hyodo and Charnoz 2017) may allow for somewhat younger rings, and hence, younger ring-born moons. Although there is still some uncertainty in the availability of these large objects over time, ring formation via tidal disruption of a passing object would have been much more likely billions of years ago (e.g., Nesvorny 2018; Nesvorny et al. 2023). In either case, the amount of time needed to assemble the mid-sized moons depends on the details of the model and how many of the moons are thought to have emerged from the rings, which also places a lower limit on the initial ring mass. Both options for ring formation produce rings early enough in Saturn’s history that the moons would have billions of years to emerge from the rings, undergo interior evolution, and develop their geologic records.

*Cassini* measurements have shown that the mass of Saturn’s rings is consistent with that of ancient rings. Over time, a disk of material around a planet will lose mass, with ∼80% of the disk’s mass falling inward onto the planet and ∼20% of the mass escaping outward, through the Roche limit, where it can assemble into moons (e.g., Salmon and Canup 2017). As the disk mass decreases, the process of material loss slows, leading to an asymptotic mass over time. The current mass of Saturn’s rings (Iess et al. 2019) is consistent with the predicted asymptotic mass for an evolved disk, a process that takes ∼2 Gyr (Salmon et al. 2010; Crida et al. 2019). Therefore, the rings are at least 2 billion years old or they just happened to form with a mass close to that of an ancient, evolved ring system. Even though an ancient ring system seems to provide an explanation for the current ring mass and the emergence of mid-sized moons, the current ring system at Saturn may not be ancient. While the idea of young rings is not new (e.g., Esposito 1986; Cuzzi and Durisen 1990; Cuzzi and Estrada 1998, and references therein), *Cassini* measurements have provided robust data that any ring formation model must now address. In particular, the brightness and composition of the rings suggest that they may be only ∼100 Myr old (Zhang et al. 2017; Iess et al. 2019).

Very young rings present a challenge for the ring-born moon scenario, particularly if all five mid-sized moons are assumed to form in this manner, because 100 Myr may not be sufficient time for the orbits to expand to their present locations (e.g., Salmon and Canup 2017), and undergo any geologic or geophysical modification of the moons that must take place. In addition, the initial mass of the ring must have been close to the present-day mass because of the limited time available for viscous spreading and mass loss, which further reduces the likelihood of moon formation and requires that the rings coincidentally formed with about the same mass as long-lived rings. Hence, the conflicting age estimates of the rings inferred by different data sets (e.g., Salmon et al. 2010; Zhang et al. 2017; Iess et al. 2019; Crida et al. 2019 and references therein) creates uncertainty as to whether emergence from rings is a viable scenario for the initial formation of the mid-sized moons.

An alternative scenario, that does not invoke rings to form the mid-sized moons, comes from Asphaug and Reufer (2013), who suggest that Saturn had an initial set of larger moons – more similar to the Galilean moons of Jupiter – that formed in the CPD. The moons underwent a series of mergers, with the collisions creating ejecta that became the mid-sized moons and the final merger producing Titan. They posit that the mergers occurred relatively early in the system’s history (roughly 3.5 Gyr ago), and that the variations in rock content across the mid-sized moons reflect different proportions of rocky core and icy mantle material that were incorporated into each moon. This model does not directly provide an explanation of the mass gradient of the mid-sized moons or how Saturn’s rings formed.

Whether mid-sized moons formed directly from the CPD, were born from ancient rings, or formed as ejecta from mergers early in Saturn system history, there would be billions of years in which the moons could then be subjected to potentially-disruptive collisions (e.g., Nesvorny et al. 2023). Several models suggest that some or all of the current mid-sized moons reassembled after collisions into or among preexisting moons (Ćuk et al. 2016; Dubinski 2019; Teodoro et al. 2023); these models are typically agnostic as to the origins of the preexisting moons. The collision and reassembly is assumed to occur within the past 100 Myr either to help explain the evolution of mean motion resonances (Ćuk et al. 2016; Teodoro et al. 2023) or to produce rings from collisional debris that are consistent with the presumed young age of the rings (Dubinski 2019; Teodoro et al. 2023). Initially, numerical models of such an event suggested that limited debris would escape from the precursor moons (Hyodo and Charnoz 2017), but more recent simulations show that both rings and substantial planetocentric debris can be generated after a collision between precursor moons (Teodoro et al. 2023). Separately, Wisdom et al. (2022) suggested that the rings formed recently via tidal disruption of a highly eccentric hypothetical moon, nicknamed Chrysalis. The model does not detail the interactions between Chrysalis and the current suite of mid-sized moons in the simulations of its migration, so we do not consider it further here.

Models that invoke disruption and reassembly imply that the entire cratering histories of the reassembled moons would have to be emplaced since the collision. The accretion of debris after the impact may account for some of the craters, as suggested by Dubinski (2019) for Mimas, but the effectiveness and geophysical implications of this mechanism have not been fully investigated. A major open question is whether reassembled moons, whether formed out of the ejecta from early mergers (Asphaug and Reufer 2013) or late collisions (Ćuk et al. 2016; Dubinski 2019; Teodoro et al. 2023), can develop interiors and geologic records consistent with the observations of Saturn’s mid-sized moons.

To summarize: 1) The ring-born moon model can explain the mass gradient of the mid-sized moons (e.g., Crida and Charnoz 2012) and relies upon a material transport mechanism that must occur (e.g., Salmon and Canup 2017), making it an appealing option for the initial assembly of moons at Saturn. Producing the mid-sized moons from the rings is more tenable if the rings are ancient (as in Charnoz et al. 2011). An important consideration is whether moons that formed billions of years ago can avoid disruptive collisions (cf., Charnoz et al. 2009; Movshovitz et al. 2015) and how collisions might have altered the very characteristics of the moons that these formation models reproduce. Although, because the moons form in sequence in these models (e.g., Crida and Charnoz 2012), with time between the emergence of each moon, it is possible that the innermost mid-sized moons were produced after the heaviest bombardment, lessening this concern. If the present rings are very young, it would be challenging to have produced the mid-sized moons via this mechanism. However, spawning moons from a previous generation of rings, an older age for the current rings, or a variant of the ring-born moon model that can produce moons and expand their orbits within ∼100 Myr may be compatible with a young ring age.

2) Models that invoke late collisions and reassembly (Ćuk et al. 2016; Dubinski 2019; Teodoro et al. 2023) may provide a pathway to generate both rings and mid-sized moons that are consistent with the inferred young age of the rings (e.g., Zhang et al. 2017; Iess et al. 2019) while relying on a process that is likely to have occurred at some point within the lifetime of the Saturn system (i.e., large collisions). Reassembly after recent disruptive collisions does not independently explain the mass gradient of the mid-sized moons. If collisional debris was preferentially reaccreted by the same precursor body (e.g., Charnoz et al. 2009; Hyodo and Charnoz 2017), characteristics of the moon that stem from its formation, such as a mass set by formation in the rings, may be preserved. However, collisional debris has also been suggested as a source of ring material and impactors that cratered the moons (Teodoro et al. 2023), implying a substantial amount of debris is lost from the precursor moon. Alternatively, a model that produces mid-sized moons from early disruptive collisions must then invoke another mechanism to generate rings (e.g., combining Asphaug and Reufer 2013 with Wisdom et al. 2022), particularly if the rings are young. Most importantly, reassembled moons must still be old enough to have developed their rich geologic records and high crater densities.

At present, models for the formation of the mid-sized moons imply ages of either billions of years or ∼100 Myr. Hence, in the following sections, we focus on geologic constraints that can help differentiate between these two end-members. It is worth noting that both ring-born moons and reassembled moons could have produced moons with intermediate ages if the processes invoked occur at different times than the present studies have explored.

## Cratering of Saturn’s Mid-Sized Moons, Insights and Limitations

### Crater-Based Ages

One of the primary ways of estimating planetary surface ages is by counting impact craters and estimating the time required to produce them, which relies upon knowledge of the impactor populations responsible for cratering. The outer planets and their satellites are predominantly cratered by Kuiper Belt objects (KBOs), Jupiter-family comets (JFCs), Centaurs, and planetocentric debris. Studies of outer planet cratering rates and/or impactor sources include: Shoemaker and Wolfe (1981), Nakamura and Yoshikawa (1995), Chapman and McKinnon (1986), Levison et al. (2000), Di Sisto et al. (2009), Di Sisto and Brunini (2007, 2011), Di Sisto and Rossignoli (2020), Di Sisto and Zanardi (2013, 2016), Rossignoli et al. (2019), Volk and Malhotra (2008), Zahnle et al. (2003), Alvarellos et al. (2005, 2017), Dones et al. (2009), Schenk and Zahnle (2007), Kirchoff and Schenk (2010, 2015), Greenstreet et al. (2015), Hirata (2016), Kirchoff et al. (2018), Singer et al. (2019), Spencer et al. (2020), Bell (2020), Ferguson et al. (2020, 2022a, 2022b, 2024), Kirchoff et al. (2022), Bottke et al. (2023, 2024), Robbins et al. (2024). It has also been proposed that ejected main belt asteroids are the primary source of impactors on the Saturn satellites (e.g., Horedt and Neukum 1984; Wagner et al. 2006; Neukum et al. 2006; Schmedemann et al. 2009), but this scenario has many challenges to overcome (see Bottke et al. 2023). Unlike the Earth’s Moon, there are no known absolute ages for the outer solar system, so crater-derived chronologies will have substantial uncertainties.

Zahnle et al. (2003), using the knowledge gained in previous works, developed two production functions and chronologies for outer solar system moons – Case A and Case B – that became the primary means, for many years, of dating cratered surfaces that were more affected by outer solar system material than by material originating in the asteroid belt. Case A was based on estimates of the contemporary impact rate of comets onto Jupiter and was tailored to match crater counts on Europa and Ganymede (Zahnle et al. 2003). Case B was formulated to provide a better match to crater counts on the surface of Neptune’s moon, Triton, under the assumption that Triton’s size frequency distribution of craters differed from that at Jupiter because the impactor flux changed with distance from the Sun (Zahnle et al. 2003). The resolution of *Voyager* images at Triton limited crater identification to D > 3 km, so the Case B production function is only tied to observations for crater sizes above that limit (Schenk and Zahnle 2007).

Overall, the impactor size frequency distribution (SFD) represented by Case A matches expectations for a heliocentric population coming from the primordial Kuiper belt that has experienced substantial collisional evolution (e.g., Bottke et al. 2023, 2024). In addition, the *New Horizons* mission acquired crater data at Pluto and Charon that is a much closer match to Case A than Case B (Singer et al. 2019; Robbins and Singer 2021; Bottke et al. 2023), casting doubt on the initial hypothesis that Triton’s SFD represented a heliocentric population that changed with distance from the Sun (Zahnle et al. 2003). Work by Schenk and Zahnle (2007) and Mah and Brasser (2019) suggested that Triton’s craters were mainly produced by planetocentric debris and possibly secondaries from unseen large craters on Triton, instead of heliocentric impactors, providing an alternative interpretation of Case B. However, the impact velocities, timing and duration of impacts, and other details of the chronology were never updated to be consistent with planetocentric impactors, so Case B cannot be used to derive ages on Triton or other bodies where planetocentric impactors dominate the cratering record. Attempts have been made to recover the characteristics of planetocentric impactor populations from crater counts on Saturn’s moons (e.g., Bell 2020), but more work is needed to attach robust ages to these empirical production functions.

We describe the crater counting studies thus far performed for the mid-sized moons of Saturn in the next section. Many of these studies, particularly those that focus on elliptical craters, support the idea that planetocentric impactors have played an important role in cratering the moons (e.g., Kirchoff and Schenk 2010; Hirata 2016; Kirchoff et al. 2018 and references therein; Ferguson et al. 2020, 2022a, 2022b, 2024; Robbins et al. 2024), even when considering updated estimates of the heliocentric impactor production function and flux (e.g., Dones et al. 2009; Di Sisto and Zanardi 2013, 2016). Hence, we refrain from presenting age estimates based on fits to existing production functions, particularly Case B from Zahnle et al. (2003), even where the cited literature does.

The real value in these crater counts is that they provide an observable population against which we can test models of possible events that produce and distribute planetocentric material to the mid-sized moons. Once the sources of planetocentric material are better constrained, we can begin estimating the timescales over which the craters were emplaced. It is important to note, however, that some studies report fitting the broad characteristics of the crater populations on Saturn’s mid-sized moons using models of exclusively heliocentric impactors (Wong et al. 2019, 2021, 2023; Bottke et al. 2024). The models estimate the surface ages of the moons to be >4 Gyr old. We consider these ages to be upper limits as the presence of planetocentric sources would add to the crater populations contemporaneously with heliocentric sources.

In addition to primary cratering, secondary and sesquinary cratering may contribute to the populations on icy moons, particularly on the lower-gravity moons around Saturn. Secondary cratering is the process in which material ejected from a primary impact onto a body is launched outwards and re-impacts the same surface as the primary crater, creating an additional crater (Bierhaus et al. 2012, 2018; Alvarellos et al. 2005, 2017; McEwen and Bierhaus 2006; Singer et al. 2013). Impact velocities for the ejecta that form secondaries are, necessarily, slower than the target’s escape velocity (McEwen and Bierhaus 2006). Mimas and Enceladus are not expected to have significant secondary crater populations due to their very low escape velocities, which approach the threshold velocity required to make a secondary crater (Bierhaus et al. 2012). Rhea, Tethys, and Dione have high enough surface gravities to retain ejected material and form secondaries (Bierhaus et al. 2012; Schenk et al. 2020).

Ejecta from a primary impact that does exceed the escape velocity of the impacted body can eventually impact either the same body or another body, depending on the dynamics of the system. Craters created by this ejecta are called sesquinaries (Alvarellos et al. 2005, 2017; Zahnle et al. 2008; Bierhaus et al. 2012, 2018), and the ejecta that forms them can be considered a type of planetocentric impactor. Sesquinaries may remain in orbit around the original moon or travel between moons, where they can contaminate the primary crater population. Alvarellos et al. (2005, 2017) used dynamical models to estimate the fraction of debris that should be present on the surfaces of the mid-sized Saturnian moons due to sesquinaries from large primary impacts onto a different moon. For the impact events that created craters such as Odysseus and Penelope on Tethys, 82–96% of ejected material came back to Tethys, while the remaining fragments were likely captured into an orbit around Saturn (Alvarellos et al. 2005). Similarly for Rhea, 91–96% of the material ejected from the Tirawa impact event would have re-impacted Rhea (Alvarellos et al. 2005). For the Herschel basin on Mimas, ∼99% of all ejected material returned to Mimas in the form of sesquinary impacts (Alvarellos et al. 2005, 2017). Most sesquinary cratering on worlds like Enceladus and Dione will have average crater diameters on a scale of 200 m or smaller (Alvarellos et al. 2017), rendering these craters far below the current image resolutions for the data from *Cassini*.

An important consideration in the interpretation of cratered surfaces is the extent of saturation. A surface is in crater saturation equilibrium when the crater density becomes high enough that, on average, a newly-formed crater erases another previously formed crater, such that the crater spatial density reaches a steady-state (e.g., Gault 1970; Hartmann 1984; Chapman and McKinnon 1986; Richardson 2009). Saturation primarily affects the computation and comparison of surface ages because a saturated surface is older than can be derived from either absolute or model-based ages. In particular, if the heavily-cratered terrains of the satellites are saturated, the surfaces could be different ages even if their relative crater spatial densities are similar. Whether the surfaces of the mid-sized moons are saturated has been debated since *Voyager* (e.g., Hartmann 1984; Chapman and McKinnon 1986; Lissauer et al. 1988; Squyres et al. 1997; Kirchoff and Schenk 2010; Kirchoff et al. 2018). Until the saturation issue is resolved, no definitive conclusions can be drawn as to the relative ages of heavily cratered regions across different mid-sized moons.

The other effect saturation equilibrium can have is modifying the shape of the crater size-frequency distribution (SFD) from its production function (Gault 1970; Chapman and McKinnon 1986; Richardson 2009). Specifically, in cases where the slope of the production function is steeper than approximately −2.5, the crater SFD would become shallower (closer to −2) when the distribution enters saturation. However, if the production slope is shallower than approximately −3, the crater SFD generally does not change significantly as a result of saturation and still represents the production function (e.g., Woronow 1978, 1985; Chapman and McKinnon 1986; Richardson 2009). Fortunately, the crater SFDs of Saturn’s mid-sized moons appear to have overall shallower slopes (Kirchoff and Schenk 2010; Bell 2020; Ferguson et al. 2020, 2022a, 2024; Robbins et al. 2024), making it likely that they are still representative of the impactor population(s) that created them.

Finally, several models have investigated how the mid-sized moons would have been affected by the intense heliocentric bombardment produced by the clearing of the primordial Kuiper belt (e.g., Gomes et al. 2005; Nesvorny 2018; Nesvorny et al. 2023; Bottke et al. 2023). For example, Nimmo and Korycansky (2012) determined that early bombardment would cause Mimas and Enceladus to lose all of their volatiles (as well as Miranda at Uranus), which does not match the high mass fractions of ice in the moons (e.g., Castillo-Rogez et al. 2018). The favored explanation of Nimmo and Korycansky (2012) was that their modeled bombardment population was roughly ten times too large. The bombardment predictions were revised downward by Bottke et al. (2024), who found the expected impact flux was a factor of 5 smaller than previously suggested, enough that volatiles on the icy worlds could survive early bombardment.

Using the original estimated mass of the early bombardment population, Charnoz et al. (2009) showed that Mimas would have been disrupted, Enceladus had a roughly 50% chance of disruption, and Tethys, Dione, and Rhea likely survived early heavy bombardment. In contrast, Movshovitz et al. (2015) found that Mimas, Enceladus, and Tethys experienced at least one catastrophic impact in every one of their bombardment simulations. Furthermore, they determined that the delivered mass of impactors would need to be a factor of 100 lower for Mimas to avoid disruption and about 30 times lower to save Enceladus. Comparable results were also found by Wong et al. (2019, 2021). Regardless of the early impactor mass, Mimas and/or Enceladus may have avoided disruption if their final assembly occurred late enough to miss peak bombardment, such as via ring formation (e.g., Salmon and Canup 2017). Given the various ways in which impacts can affect mid-sized moons, it is plausible that the current suite of mid-sized moons experienced major changes in their bulk properties since their formation, whether they formed in Saturn’s CPD, emerged from ancient rings, or formed from early mergers.

### Overview of Crater Counts in the Saturn System

**Fig. 2:**
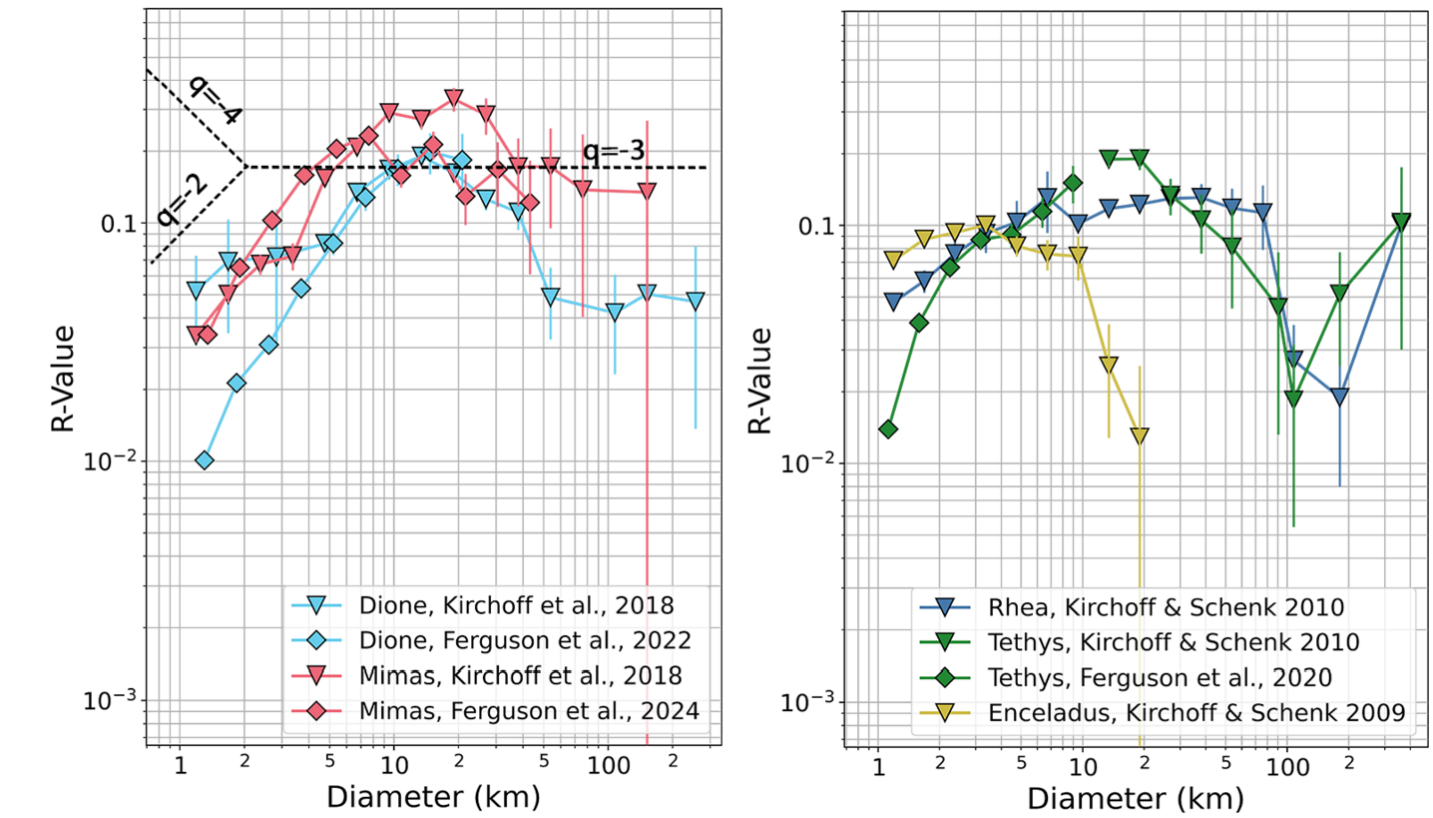
Crater distributions shown on an R-plot, on which a horizontal line would correspond to a −3 slope on a log-log SFD plot (see dotted lines in the left panel). Data for each moon is shown in a different color, with symbols representing the crater counting study from which the data was taken. For Tethys, we show results from Kirchoff and Schenk (2010) only for craters larger than ∼10 km because the size and locations of the areas mapped make the counts less complete than the Ferguson et al. (2020) data set

The study of impact cratering on the mid-sized moons has a long history, dating back to the earliest exploration of the outer solar system. *Voyager*-era studies, and their implications, are described in detail in Kirchoff et al. (2018), along with the post-*Cassini* view up to that point. As impact cratering is only one of the geologic feature types we discuss in this work, we summarize the major characteristics of the cratering records of the moons, with an emphasis on work that post-dates Kirchoff et al. (2018). Examples of crater data for the moons are shown on the relative plots (R-plots) in Fig. 2, in which the colors represent the moons, and the symbols represent the different studies where the data was presented. The data shown from Kirchoff et al. (2018) combines several different datasets (Kirchoff and Schenk 2009, 2010, 2015; Robbins et al. 2015).

The value of an R-plot is that it more clearly separates data with log-log SFD slopes (labeled $q$ on Fig. 2, left panel) that fall between −2 and −4, which applies to many surfaces throughout the Solar System (Crater Analysis Techniques Working Group, 1979). As indicated in Fig. 2 (left panel), data that plots as a horizontal line on an R-plot would have a slope of −3 on a log-log SFD plot. When comparing data on R-plots, the vertical placement of the curve indicates the overall number of craters normalized by area, in which higher R values correspond to a higher spatial density of craters and, in the simplest case, indicate a longer exposure age. Because the data is Fig. 2 has not been scaled to account for differences in surface gravity, impactor speed, or the expected increase in the number of impactors with proximity to Saturn across the mid-sized moons, the vertical placement of the data cannot be used to infer anything about the relative ages of the moons. The slopes of distributions on an R-plot represent the shapes of the impact populations that created them. Breaks in slope (i.e., going from a shallow slope to a steeper slope) may indicate a change in the impactor population at the diameter value where the break in slope occurred. If the slope of the impactor production function is well-known, deviations could indicate that multiple impact sources have affected the surface and/or the surface lost craters due to other types of geologic resurfacing.

The shapes of the crater distributions on Mimas and Dione are similar between the data sets shown in Fig. 2 (left panel; pink and blue, respectively), with a sharp drop in crater density at small sizes (D < 5 km) and a modest drop for larger craters (D > 40 km). For Tethys (right panel; green), we show crater data from Ferguson et al. (2020) for smaller craters and Kirchoff and Schenk (2010) for larger craters, based on the estimated completeness diameters of each data set. In particular, Kirchoff and Schenk (2010) also reported data for the smaller size range of craters on Tethys, which differ from the Ferguson et al. (2020) results due to differences in the sizes and locations of the count areas, but those counts are not as complete. For craters with diameters of 1–10 km, Tethys’ craters follow trends fairly similar to those on Mimas and Dione, while for the larger craters (20–100 km), Tethys’ distribution shows a more significant drop in crater density. The crater distribution on Rhea (right panel; blue) is flatter than those on Mimas, Tethys, and Dione until ∼70 km where the crater density drops in a similar fashion to the Tethys data. Enceladus shows a flatter distribution for small craters (<10 km), but drops off rapidly for craters larger than 10 km (right panel, yellow).

Comparisons between the moons show that the heavily cratered regions of Mimas, Tethys, and Dione have size frequency distributions more similar to each other than to Rhea or more distant Iapetus (Kirchoff et al. 2018). For smaller impact craters (D < 5 km), Mimas has a higher overall crater density than Tethys, which has a higher crater density than Dione (Ferguson et al. 2020, 2022a, 2024). In contrast, there are some indications that Mimas lacks impact craters with diameters ∼30–80 km relative to the other mid-sized moons (Kirchoff and Schenk 2010). Using relative spatial densities of impact craters ∼10 km and larger, which are harder to remove by subsequent resurfacing than smaller craters, Ferguson et al. (2024) found that Dione has preserved the oldest surfaces, followed by Tethys, and then Mimas. However, all three moons appear to be within about a factor of two of one another in age. For the largest craters sizes (D > 100 km), the cumulative number of impact craters per square kilometer on the most ancient terrains of Mimas, Tethys, Dione, and Rhea are fairly comparable to one another (Kirchoff and Schenk 2010). Although there are sometimes variations in the results of crater counting studies, there is good agreement across the Mimas data sets, and Rhea consistently shows a relatively flat slope across a wide range of impact crater sizes and some variation between the leading and trailing hemispheres (Robbins et al. 2024).

On Enceladus, crater spatial densities are highly variable due to geologic resurfacing (e.g., Crow-Willard and Pappalardo 2015; Kinczyk et al. 2022). Overall the crater density is lower than the other moons, particularly for impact craters smaller than 2 km, and Enceladus lacks larger impact craters (∼6 km and larger) when compared to the other satellites in the system (Kirchoff and Schenk 2009). The greatest numbers of impact craters are on the sub- and anti-Saturn hemispheres (e.g. Kirchoff and Schenk 2009) rather than the leading and trailing hemispheres where tectonic resurfacing has erased most of the early cratering record. Impact craters may have also been removed through a combination of viscous relaxation and mantling by plume fall-back (Bland et al. 2012) as evidenced by heavily modified craters, especially in the northern hemisphere (e.g., Bland et al. 2024). The sparseness of impact craters on Enceladus’ surface is further highlighted when compared against the heavily cratered terrains of Rhea and Tethys (Fig. 2, right panel, yellow). Enceladus’ craters show a different slope from the other two moons, and the crater density drops for craters larger than ∼4 km, which suggests erasure by various resurfacing and surface modification mechanisms that are active on Enceladus (Kirchoff and Schenk 2009).

Impact basins provide additional benchmarks with which to assess surface ages of a moon. Here, we focus on the Odysseus basin (D = 445 km) on Tethys and the Herschel basin (D = 139 km) on Mimas. By assessing the crater distributions both within and outside of the basins, the Odysseus impact basin was shown to be ∼4–9 times younger than heavily-cratered terrains on Tethys (Kirchoff and Schenk 2010). For the Herschel basin on Mimas, Ferguson et al. (2024) showed that the ejecta blanket and interior have far lower crater densities than the most heavily cratered regions (i.e., around Mimas’ north pole), suggesting that Herschel formed within the most recent ∼10-20% of Mimas’ surface age. Hence, large impacts can occur even relatively recently in the lifetimes of the mid-sized moons.

Based on the cratering records across Saturn’s moons, Kirchoff and Schenk (2010) hypothesized that Mimas, Tethys, and Dione record mainly a planetocentric population of impactors, as evidenced by their steeper-sloped SFDs that indicate an abundance of small craters relative to larger ones. More recently, Bell (2020) compared the crater populations across Saturn’s moons, using existing crater catalogs and scaling laws, and found similar crater densities from Mimas through Rhea, suggesting a common impactor source. When compared with predictions using heliocentric (using the model of Di Sisto and Zanardi 2013) or planetocentric impactors, Bell (2020) concluded that the moons experienced predominantly planetocentric cratering, even when crater saturation was considered.

At Mimas, the Case B production function provides an excellent fit to the SFDs in all mapped regions (Ferguson et al. 2024), for impact craters larger than a few km, supporting a planetocentric origin. Case A can match the slope of the SFDs for smaller impact craters within one heavily cratered region, but it vastly overpredicts the number of larger impact craters as compared with the observations. Within the Herschel basin and the ejecta blanket, which have a lower spatial density of craters overall, Case A and Case B can both match large portions of the SFD. However, surface ages derived from Case A fits (which are tied to the more robust heliocentric chronology) produce unrealistic results, in which the interior and ejecta blanket of Herschel are estimated to be billions of years older than the heavily cratered regions of Mimas. While it’s possible that the improvement in the fit of Case A within regions of Mimas with lower crater densities indicates a change to a more balanced mix of heliocentric and planetocentric impactors, the unphysical ages add skepticism (Ferguson et al. 2024).

Similarly, at Tethys and Dione, Case B provides a much better fit to the data at impact crater diameters ∼3–10 km and larger (depending on region) than Case A, although the fits are not as good as those at Mimas, perhaps indicating differences in the planetocentric flux across the mid-sized moons (Ferguson et al. 2020, 2022a, 2024). Again, Case A only fits the SFD slopes at small diameters and overpredicts the number of larger impact craters due to the slope of the production function. At Rhea and Iapetus, Kirchoff and Schenk (2010) posit that the impact craters are more representative of the heliocentric population, although Hirata (2016) suggests that – for Rhea – only D > 20 km craters are predominantly heliocentric in origin, due to the presence of an asymmetry in the crater distribution at that size between hemispheres.

### The Distribution and Implications of Elliptical Craters

**Fig. 3:**
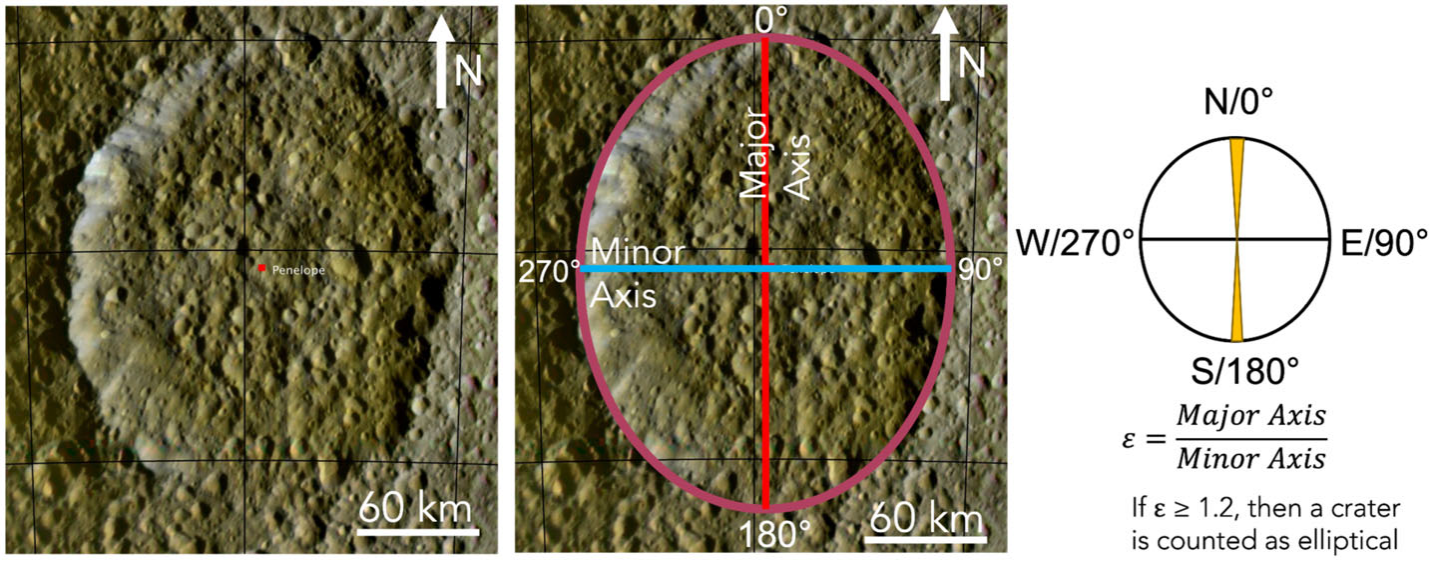
Penelope crater on Tethys (left) is elliptical rather than circular, which indicates a relatively slow, oblique impact. Here, we show the major and minor axes of Penelope (center), the relationship between “orientation” and the azimuth of the major axis (right), and the equation for determining the ellipticity of a crater.

Recent work mapping and analyzing elliptical impact craters on the mid-sized moons may provide some additional constraints on the scope and nature of planetocentric debris in the Saturn system. Elliptical impact craters (Fig. 3) are particularly useful for characterizing an impactor population because the long axis of an elliptical crater records the impact direction, and the formation of an elliptical, rather than circular, crater implies a relatively low impact angle (Gault and Wedekind 1978; Bottke et al. 2000; Collins et al. 2011; Elbeshausen et al. 2013; Holo et al. 2018).

**Fig. 4:**
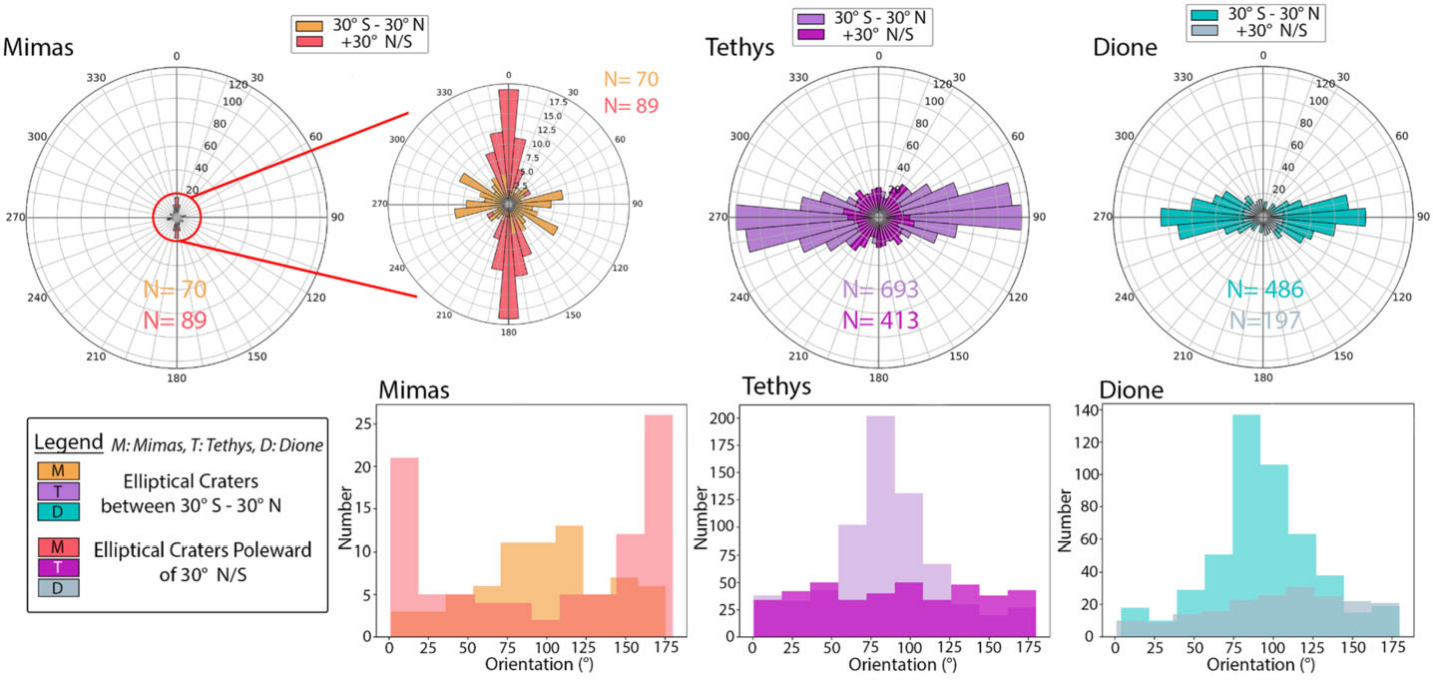
Two styles of histogram show the orientations of the long-axes of elliptical craters on Mimas, Tethys, and Dione, in which the populations are split by latitude. Across all three moons, regions within 30° of the equator contain elliptical craters that are predominantly oriented east-west, although the signal is less apparent on Mimas. On Tethys and Dione, this group makes up the majority of the elliptical craters, with a smaller, more isotropically-oriented group spanning all mapped latitudes. On Mimas, elliptical craters above 30°N are predominantly oriented north-south; this population appears to be unique among the three moons. Image credit: Ferguson et al. (2024)

On Mimas, Tethys, and Dione, elliptical impact craters with diameters between 2 and 70 km have been mapped as globally as possible within the existing image data (Ferguson et al. 2022b, 2024). Mimas and Tethys have a similar spatial density of elliptical craters, with the more heavily resurfaced Dione displaying a somewhat lower spatial density (Ferguson et al. 2024). Tethys and Dione display a remarkable pattern in which the majority of elliptical craters have their long axes oriented east-west (Fig. 4) and are concentrated in an equatorial band between 30°N and 30°S (Ferguson et al. 2022b). At Mimas, the equatorial east-west oriented population is not as apparent (Fig. 4), perhaps because the Herschel-forming impact erased existing elliptical craters along the equator (Ferguson et al. 2024). Tethys and Dione also display smaller populations of elliptical craters with long axis orientations that are isotropic, which appear to be globally-distributed; this population is not observed on Mimas. Instead, Mimas’ elliptical craters become more azimuthally clustered far from the equator, and the dominant orientation is north-south rather than east-west.

One potential source for the elliptical impact craters on Tethys and Dione is a planetocentric debris disk that flattened over time. A debris disk could form after a large impact, as explored by Hyodo and Charnoz (2017), although more recent models suggest a significant fraction of collisional debris may be lost to Saturn instead of impacting the moons (Teodoro et al. 2023). Because the trajectories of debris tend to become more uniform as a disk flattens (e.g., Brahic 1977), this mechanism may explain both the isotropically-oriented global population and the east-west equatorial population (Ferguson et al. 2022b). Currently unknown is whether the moons were impacted with material from a common debris disk or experienced impacts that generated independent debris disks. Tethys and Dione have similar diameters and similar latitudinal extents to their east-west elliptical crater populations, suggesting that the source of impacting material had the same thickness at the locations of each moon. Curiously, Mimas has a similar band of east-west elliptical craters as Tethys and Dione, despite its smaller diameter, and it lacks an isotropically-oriented population. Either Mimas was exposed to a local debris disk with different characteristics, or a common debris disk affected all the moons but had already flattened considerably when it reached Mimas.

An important consideration is that both Mimas and Tethys currently have orbital inclinations that take them out of Dione’s orbital plane (Table 1). In order for the moons to be subjected to the same debris disk and have similar latitudinal patterns, the elliptical craters had to be emplaced before the inclinations of Mimas and Tethys were raised. The uncertainties in the long-term dynamical evolution of the moons (e.g., Ćuk et al. 2016; Ćuk and El Moutamid 2022; Nakajima et al. 2019) make this scenario plausible and may provide constraints on the history of mean motion resonances among the moons. Alternatively, the similarities across the moons may indicate a common process of impact-generated debris and accretion that affected each of the three moons independently (Ferguson et al. 2022b).

The non-circular nature of elliptical impact craters makes it challenging to interpret their SFDs with the standard crater analysis tools that were developed for circular craters. First, there is the issue of crater diameter, which varies over the crater’s perimeter. If only elliptical craters are being included in the SFD, it may be reasonable to simply apply a consistent rule, such as using the geometric mean of the major and minor axes of each elliptical crater (as in Ferguson et al. 2022b). However, mixing elliptical and circular craters on the same SFD is problematic as the scaling laws that connect impact parameters with elliptical crater dimensions on icy surfaces are still being developed (e.g., Elbeshausen et al. 2013). While we know that the impact angle controls whether the impact forms a circular or elliptical crater, how the dimensions of the elliptical crater change with impact angle is still under investigation. Thus, we cannot tell which circular and elliptical craters were formed by impacts with similar impact velocities and impactor sizes.

The second challenge in interpreting the SFDs of elliptical craters is that the production functions were developed assuming impact angles that create circular craters. Again, our lack of detailed knowledge as to how lower impact angle events manifest in the dimensions of elliptical craters means that we cannot simply compare the predicted crater sizes from a production function to the crater populations on the moons. More modeling and/or experimental work that fully connects the shapes of elliptical craters on icy bodies with the dynamics and physical characteristics of the impactor (e.g., Collins et al. 2011; Elbeshausen et al. 2009, 2013) would be highly valuable.

### Linking Craters with Thermal History

**Fig. 5:**
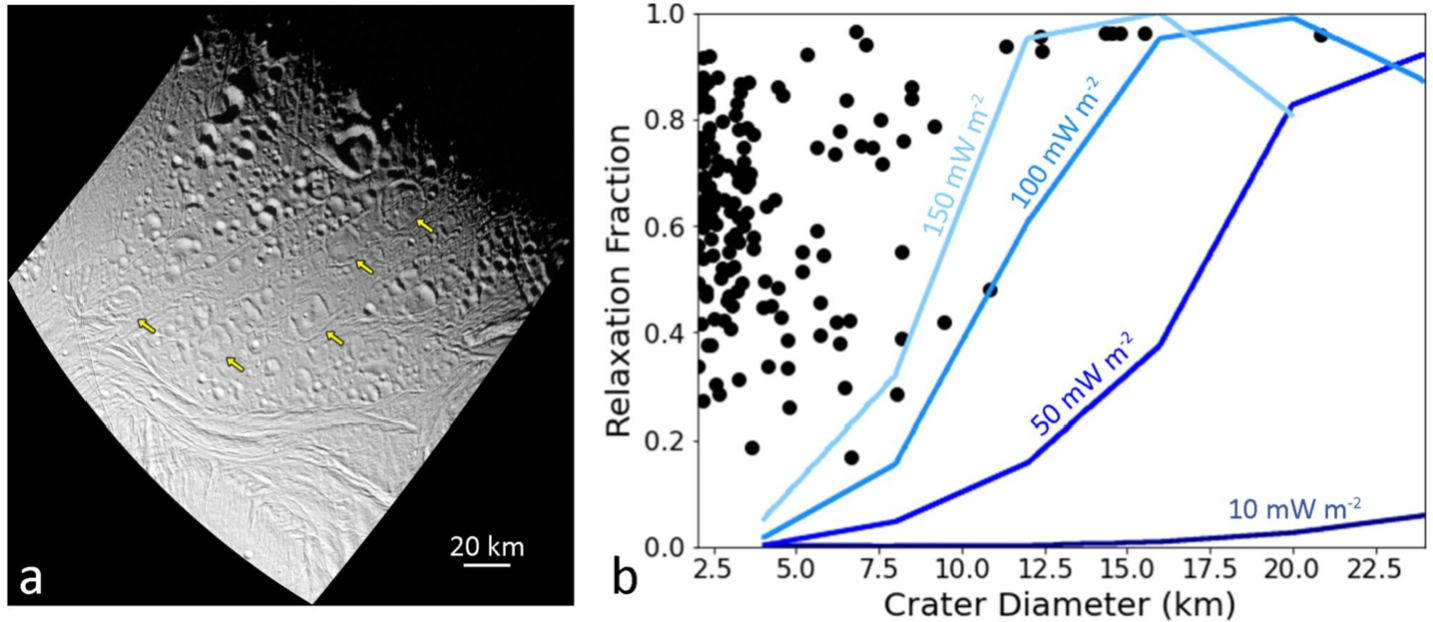
a) *Cassini* ISS image of Enceladus showing numerous viscously relaxed impact craters (yellow arrows, but many more occur throughout the area). Image N1487299402_1 in a local orthographic projection with north up. b) An example of combining observations (black points are measured crater depths on Enceladus relative to their expected depth) with numerical modeling (blue curves) to constrain the heat flux. The simulations shown here assumed a surface temperature of 120 K and a pure ice (non-porous) rheology and thermal conductivity. The modeling suggests a flux in excess of 150 mW m^−2^ was necessary to viscously relax Enceladus’ craters. Panel ‘b’ modified from Bland et al. (2012)

In addition to the spatial distribution of impact craters, their morphologies can be used to record the thermal history of a moon. Impact crater topography induces deviatoric (non-hydrostatic) stresses in the lithosphere (e.g. Dombard and McKinnon 2006). If the viscosity of the ice is low enough, these stresses can drive viscous flow that reduces the crater topography (e.g., Scott 1967; Johnson and McGetchin 1973). As the topography is reduced, the driving stress is removed and viscous flow ceases, resulting in a preserved crater rim and flat or even up-bowed floor (Fig. 5; Scott 1967; Parmentier and Head 1981). Elastic flexure enhances relaxation but is a secondary effect (Dombard and McKinnon 2006).

The viscosity of rock and ice is strongly temperature dependent (e.g., Karato and Wu 1993; Durham and Stern 2001). Thus, determining whether crater topography has been modified by viscous relaxation provides a constraint on the thermal conditions the crater has experienced. Furthermore, because the viscous relaxation process also depends on the wavelength of the topography (long wavelengths are reduced faster than short ones; Parmentier and Head 1981), determining the diameter range over which craters have viscously relaxed provides a lower bound on the thermal conditions (e.g., small craters require warmer conditions to viscously relax). Thus, the thermal history of an icy satellite can be constrained by combining robust numerical modeling of viscous relaxation (e.g., Dombard and McKinnon 2006) with observations of crater depths (e.g., Passey 1983; Bland et al. 2012, 2017, 2023; White et al. 2013, 2017; Singer et al. 2018; Bland and Bray 2024).

While viscous relaxation provides critical insight into the thermal history of icy moons, it also faces several challenges. The observation that an impact crater’s topography is subdued only indicates that the viscosity of the lithosphere was low enough for relaxation to occur. The viscosity depends on both temperature and composition and is therefore non-unique. Although pure H_2_O ice is often assumed for simplicity, the lithospheres of icy moons may include other materials, including salts, dusty particulates, or ice clathrates that increase its viscosity (e.g., Friedson and Stevenson 1983; Durham et al. 1992; Mangold et al. 2002; Durham et al. 2010; Qi et al. 2018), or ammonia and ammonia hydrates that reduce it (Durham et al. 1992). The latter species has been tentatively detected from ground-based observations of Enceladus (Emery et al. 2005; Verbiscer et al. 2006) and Tethys (Verbiscer et al. 2008) and *Cassini* Visible and Infrared Mapping Spectrometer observations of Dione (Clark et al. 2008) and Iapetus (Clark et al. 2012).

Even if the composition can be assumed, the temperature structure of the lithosphere is a function of surface temperature ($T_{s}$), heat flux ($F$), and thermal conductivity ($k$). Assuming a temperature dependent thermal conductivity of the form $k = C/T$, where $T$ is the temperature and $C$ is a constant (e.g., Petrenko and Whitworth 1999, state $C = 651\text{ Wm}^{-1}$, whereas Carnahan et al. 2021, find $C = 612\text{ Wm}^{-1}$), the temperature as a function of depth, $z$, is given by

$$ T \left ( z \right ) = T_{s} \exp \left ( \frac{Fz}{C} \right ). $$

The surface temperature, $T_{s}$, is a function of surface albedo and latitude, can be modified by an insulating regolith (Passey and Shoemaker 1982) or solid-state greenhouse (Brown and Matson 1987; Matson and Brown 1989), and may have changed over the moon’s history due to substantial changes in surface albedo, especially for low-albedo moons like Callisto or Iapetus, and a weaker young Sun (Gough 1981; Bahcall et al. 2001). The thermal conductivity of pure ice is well known, but it can be modified by the inclusion of other species. For example, clathrates (English and Tse 2009) and ammonia-doped water ice (Lorenz and Shandera 2001) have thermal conductivity 2–3 times lower than water ice. Even for pure water ice, if the upper portion of the lithosphere is porous (e.g., Eluszkiewicz 1990; Kossacki and Leliwa-Kopystyński 1993; Leliwa-Kopystyński and Kossacki 2000; Besserer et al. 2013), the thermal conductivity will be substantially reduced (Shoshany et al. 2002). Thus, the bounds on the lithospheric heat flux, $F$, itself must be carefully qualified because the temperature structure $T(z)$ is a non-unique combination of $F$ and $k$. Furthermore, in many cases the same subdued topography can result from either a high heat flux imposed over a short timescale (e.g., a pulse of heat) or a lower heat flux imposed over a long timescale (e.g., Bland et al. 2017), so additional information must be used, such as the moon’s dynamical history, to reduce the uncertainty.

Inferring heat flows from crater shapes also requires knowledge of the original dimensions of the crater, which can vary based on the material properties at the surface, the thermal profile of the ice at the time of impact, whether the ice shell is conductive or convective, and whether there is an ocean under the ice (Turtle and Pierazzo 2001; Senft and Stewart 2011; Bray et al. 2014; Silber and Johnson 2017; Denton and Rhoden 2022; Bjonnes et al. 2022). Although the original crater dimensions are often inferred by measuring fresh-appearing craters on the moon’s surface, in some cases relaxation occurs rapidly enough under relatively quiescent conditions that even these depths are too shallow (Bland and Bray 2024). Incorrect inference of the original crater depth leads to an inaccurate estimate of both the degree to which the crater has relaxed (e.g., the crater may simply have formed shallow), and the timescale over which relaxation has occurred. For a stress-independent rheology, the relaxation timescale (i.e., the e-folding time) is independent of crater depth; although relaxation occurs faster for a deep crater than a shallow one due to the larger stresses involved, greater vertical displacement of the crater floor is also required (Turcotte and Schubert 2002). However, the viscosity of icy material is highly stress-dependent (for a summary, see e.g., Durham et al. 2010) and deep initial craters in ice therefore relax more rapidly than shallow ones. In other words, under the same conditions, a deeper initial crater will have a greater relaxation fraction than an initially shallower crater.

Despite these challenges, crater shapes can still be used to draw inferences as to the past heat flows on icy moons, as described for each moon within Sect. 5. In addition, numerical tools to model the formation and modification of craters – particularly for the size of Herschel and for moons with subsurface oceans – have also greatly improved in recent years (e.g., Silber and Johnson 2017; Denton et al. 2021; Denton and Rhoden 2022; Bjonnes et al. 2022). These tools provide an opportunity to better predict initial crater shapes and determine more precise relaxation fractions in future studies.

## Interior Structure Characterization, Approaches and Observations

### Rotational Dynamics

Here, we briefly describe a valuable observational tool, in which the rotational motion of a moon is used to infer its internal mass distribution. Details of the theoretical approach are provided by Hemingway et al. (2018); see also Rambaux et al. (2010), Rambaux (2014), and Nimmo (2018). Results obtained for the mid-sized moons are summarized, here, while the interpretations in context with their overall geologic histories are covered in Sect. 5.

Most moons of the giant planets maintain a 1:1 spin-orbit resonance, in which the moon completes one rotation about its spin axis in the same amount of time that it completes one orbit around the planet (e.g., Peale 1976). In this case, averaged over an orbit, the same face of the moon is always pointed toward the planet, which leads to an elongation of the moons’ physical shape in the direction of the planet (i.e., the tidal axis). Moons in this configuration are described as being in synchronous rotation. Although long-period non-synchronous rotation (NSR) has been suggested for several ocean-bearing icy moons from geological interpretations, it typically refers only to the ice shell’s rotation relative to the interior, which may still be tidally locked (e.g., Greenberg et al. 1998; Patthoff and Kattenhorn 2011; Collins et al. 2010). Whether NSR is likely from a dynamical perspective is still debated (e.g., Greenberg and Weidenschilling 1984; Bills et al. 2009; Goldreich and Mitchell 2010). For simplicity, we neglect this type of NSR in the present discussion.

When a synchronously-rotating moon has an eccentric orbit, the moon’s orbital speed will increase and decrease as the moon moves through the closer (pericenter) and farther (apocenter) parts of its orbit, respectively. The moon’s spin rate and orbital rate will, therefore, be equal *on average* but not instantaneously throughout the orbit. As a result, the long axis of the moon will oscillate back and forth relative to the direction of the planet. Such motion is referred to as the optical libration because it is related to the relative velocity of spin and orbit. The misalignments between the long axis of the moon and the planet, associated with the optical libration, will lead to torques that also generate oscillations; these oscillations are called physical librations, and they are the dynamical response of the moon to the gravitational torque of the planet. The amplitudes of the short period physical librations are tied to the interior structure of the moon through the moment of inertia. The largest libration is the diurnal libration (libration at orbital period); its amplitude can reveal whether a moon is differentiated and, in some cases, reveal the presence of an ocean.

Libration can be measured by tracking the subtle longitudinal motion of surface features within images taken at different times in a moon’s orbit. The changes in location resulting from libration can be small – of order 100s of meters for Enceladus (Thomas et al. 2016), for example. Hence, identifying the libration signature requires (at a minimum) precise knowledge of the moon’s orbit and rotation, the spacecraft’s position/pointing, and the locations of surface features. Once the libration has been measured, its value is compared with models of the librations, derived using different plausible interior structures and the measured shape of the moon. These models typically use simplified structures in which the body is represented as nested, uniform layers; the number of layers, the layer radii, and the layer densities are then varied to capture the parameter space.

*Cassini* images provided sufficient coverage to measure librations of Mimas (Tajeddine et al. 2014) and Enceladus (Thomas et al. 2016; van Hoolst et al. 2016; Park et al. 2024). The Mimas results require that the interior is differentiated, but the data could be fit by either a subsurface ocean or a frozen outer layer over an elongated core (Tajeddine et al. 2014). As described in Sect. 5.2, additional studies of Mimas’ librations and other dynamical properties favor the ocean model (Caudal 2017; Noyelles et al. 2019; Lainey et al. 2024). Enceladus’ libration is only compatible with a differentiated interior and global sub-surface ocean (Thomas et al. 2016). Complications of the data collection and interpretations using *Cassini* data are described in Hemingway et al. (2018) as well as in the papers presenting libration measurements (Tajeddine et al. 2014; Thomas et al. 2016).

### Shape and Gravity

Here, we summarize the approach of gravity and shape measurements for inferring the interior structure of a moon. For a more detailed treatment, including the main equations, we refer the reader to Hemingway et al. (2018). We also provide a summary of *Cassini* gravity measurements of the mid-sized moons, and their interpretations. Additional discussion of these findings is provided within the individual moon sections (Sect. 5).

For a fluid moon in synchronous rotation around its parent planet, the moon will adopt a triaxial ellipsoidal shape with the associated gravity field. However, because a moon is likely to increase in density with depth, its physical responsiveness to deformation deviates from that of a uniform fluid sphere, so the triaxial shape and gravity field include the scaling factors, $h_{2f}$ and $k_{2f}$, which are the respective degree-2 fluid Love numbers. The scaled shape and gravity field can then be described using weighted mathematical functions (i.e., spherical harmonics), where the weights are $H_{lm}$ and $C_{lm}$, respectively, and $l$ and $m$ relate the weight to its function. This approach is qualitatively similar to describing a color in terms of the percentage of red, yellow, and blue that – when mixed – can produce it; the percentages act as weights while the primary colors represent orthogonal “basis” functions. By matching observational data of a moon’s shape and gravity field, the values of the weights can be constrained.

If there has been sufficient time and/or heat, the shape of a moon will conform to an equipotential surface determined by the body’s gravity, rotation, and tidal deformation. In this case, the moon is considered to be in hydrostatic equilibrium, and the ratios of the degree-2 shape and gravity weights, $-\text{H}_{20}/\text{H}_{22}$ and $-\text{C}_{20}/\text{C}_{22}$, will be 10/3. By convention, $-\text{C}_{20}$ is referred to as J_2_, such that $\text{J}_{2}/\text{C}_{22} = 10/3$ for a hydrostatic gravity field, although slight corrections may also be required for fast rotating bodies (see Tricarico 2014). These ratios provide a measure of how close (or far) the moon is to a hydrostatic shape or hydrostatic gravity field. Critically, if a moon is in hydrostatic equilibrium, H_20_, H_22_, J_2_ and C_22_ can be directly related to the polar moment of inertia, C, which constrains the density structure of the moon. In particular, $\text{C/MR}^{2} = 0.4$ for an undifferentiated moon, where M is the mass and R is the mean equatorial radius. Lower values indicate a more centrally-condensed interior, suggesting a differentiated moon.

If the moon is not in hydrostatic equilibrium, more complicated strategies must be employed to constrain the interior. Typically, the hydrostatic and non-hydrostatic components of gravity and shape are assumed to be separable from one another. Then, using multiple models of the interior structure of the body, the model parameters (such as radius and density of each layer) that best fit the measured gravity and shape can be determined (e.g., Iess et al. 2014; Tortora et al. 2016; Zannoni et al. 2020). Non-hydrostatic components may be related to uncompensated surface topography, which would suggest a relatively cold and rigid exterior. They could also be the signature of density variations within the body such that the assumption of uniform layers is invalid, or a core shape that is not hydrostatic. This last option may be common to moons that have low central pressures or may be an indication that the interior experienced insufficient heating to relax the core shape (e.g., Castillo-Rogez et al. 2018; Hemingway et al. 2018).

For the mid-sized moons, the Cassini spacecraft collected sufficient limb images (for shape) and Doppler tracking data (for gravity) to determine H_20_/H_22_ and J_2_/C_22_ for Enceladus (Iess et al. 2014; Hemingway et al. 2018 and references therein; Park et al. 2024), Dione (Thomas 2010; Nimmo et al. 2011; Zannoni et al. 2020), and Rhea (Tortora et al. 2016). Detailed descriptions of the results are included in the individual moon section. These studies show that the shapes of Enceladus and Dione are strongly non-hydrostatic, while the larger uncertainty at Rhea makes the results inconclusive. In terms of gravity, Dione and Rhea are significantly non-hydrostatic, while Enceladus has a more modest deviation from hydrostaticity. For both Enceladus and Dione, the gravity field is closer to hydrostatic equilibrium than their shape, which is interpreted as the result of a sub-surface ocean enabling compensation of overlying topography (e.g., Iess et al. 2014; Beuthe et al. 2016; Zannoni et al. 2020). In contrast, the large uncertainty in Rhea’s shape makes it compatible with hydrostatic equilibrium, so the non-hydrostatic gravity is interpreted as a limited excess oblateness of a core, which implies differentiation of the moon (Tortora et al. 2016). There is no gravity data at Mimas or Tethys; estimates from their global shapes suggest that neither moon is in hydrostatic equilibrium (Thomas 2010).

## The Geology of the Mid-Sized Moons

### Mimas

#### Overview of Surface Geology

**Fig. 6:**
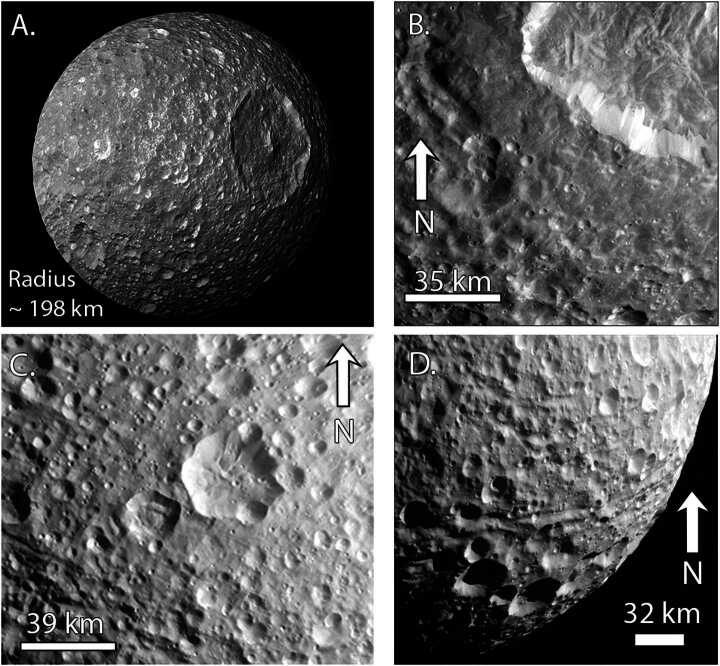
Views of Mimas across its leading and trailing hemispheres. A) Full disk view of Mimas with a focus on the Herschel impact basin (D = 139 km) and its central peak. Image PIA12570. B) Close-up of Herschel’s ejecta blanket. The lower density of craters on the ejecta blanket suggests relatively recent formation of the basin (Ferguson et al. 2024). Image N1644778567_1. C) Trailing hemisphere cratered terrain along with some grooves. Image N1831441742_1. D) Oblique view of the grooves on Mimas’ trailing hemisphere, which are among the only tectonic features so far identified on Mimas. Image N1831443018_1

Mimas’ geologic record is dominated by craters (Fig. 6), including the Herschel impact basin (D = 139 km). As discussed in Sect. 3.2, the Case B production function from Zahnle et al. (2003) provides an excellent fit to much of the crater data at Mimas (Ferguson et al. 2024), providing further support for planetocentric impactors (Kirchoff and Schenk 2010; Hirata 2016; Kirchoff et al. 2018; Bell 2020). Cratering is more sparse within the Herschel basin and on its eject blanket than on other regions of Mimas, implying a young relative age for the basin (Ferguson et al. 2024). Using the relative crater spatial densitites at Mimas, Tethys, and Dione, Ferguson et al. (2024) determined that heavily-cratered regions of Mimas are younger than those on Tethys and Dione. However, the relative ages are all within a factor of 2–3 of each other. Hence, Mimas cannot be of order 100 Myr old unless Tethys and Dione are as well, but if Mimas is at least a billion years old, Dione could be 2–3 billion years older.

As described in Sect. 3.4, crater morphologies can record the extent of heating over time. Using Voyager data, Schenk (1989) concluded that Herschel is unrelaxed. Because large craters relax more quickly than small craters, this result implied limited crater relaxation at Mimas, overall. Post-*Cassini* analyses of Mimas’ crater relaxation have been sparse. Using photoclinometry, White and Schenk (2011) analyzed the depth-to-diameter ratios of nine craters on Mimas, which were consistent with limited relaxation. Unpublished digital elevation models (DEMs) show that many deep craters, including Herschel, exist on Mimas (Schenk, pers comm), but the extent of relaxation that is compatible with the observed depths is not yet known. In addition, moons can display both heavily relaxed and unrelaxed craters due to differences in crater ages or location-based variations in heat flow, as seen on Enceladus, Tethys, and Dione (Bland et al. 2012; White et al. 2017; see also, Sects. 5.3 and 5.4), motivating a more global study. Revisiting the crater morphologies of Mimas, using the full *Cassini* data set and our improved understanding of the effects of a moon’s thermal profile and interior structure on the initial crater shape used in such studies (see Sect. 3.4), is critical for evaluating the extent of modification of Mimas’ craters.

Mimas displays little tectonic activity, particularly when compared with the other mid-sized moons, and shows no evidence of plumes or cryovolcanic flows (Schenk et al. 2018, and references therein). Arcuate grooves of uncertain origin have been identified (Fig. 6D), extending 10s of km across the surface, which may be ∼10 km deep (Schenk 2011; Schenk et al. 2018). Given that they are sometimes overprinted by craters, the grooves are probably not the youngest features on the surface. Mimas displays no evidence of tidally-driven activity akin to that observed on Enceladus (Rhoden et al. 2017; Rhoden 2023), despite a high eccentricity and close orbit around Saturn.

#### Interior Structure and Evolution

Mimas’ low density of 1.150 g/cm^3^ suggests an interior dominated by ice, as much as ∼91% in volume or ∼73% in mass, if there is no porosity (assuming a dry rock density of 3.1 g/cm^3^ consistent with a CI chondrite composition, Castillo-Rogez et al. 2018). The fraction of porosity is unconstrained, but it would be ∼30% if Mimas has an average grain density similar to that of the Uranian moons (1.6 g/cm^3^; ssd.jpl.nasa.gov). A better estimate of Mimas’ porosity could provide constraints on its formation and evolution, such as the extent of global-scale melting (e.g., Kossacki and Leliwa-Kopystyński 1993; Leliwa-Kopystyński and Kossacki 2000).

Simultaneously matching *Cassini* measurements of Mimas’ librations and precession of its orbit requires a differentiated interior and a present-day subsurface ocean (Tajeddine et al. 2014; Noyelles 2017; Caudal 2017; Lainey et al. 2024). The overlying ice shell thickness is constrained to be 20 to 30 km thick at present (Tajeddine et al. 2014; Lainey et al. 2024). If the ice shell is currently thicker than ∼29 km, it should be actively melting because there is more tidal heat than can be conducted out of the shell (Rhoden and Walker 2022). Even the thinnest shells allowed by the libration and precession measurements may be actively melting, depending on the heat flow entering the ice shell from below. Heat sources include (likely low) radiogenic heating (e.g., Hussmann et al. 2006) and tidal dissipation in Mimas’ core, which could be substantial if the core is porous (Roberts 2015; Choblet et al. 2017; Rovira-Navarro et al. 2022; Rhoden et al. 2024). Tidal dissipation within Mimas would act to circularize its orbit, particularly in the absence of a mean motion resonance (cf., Io-Europa; Peale et al. 1979), providing constraints on Mimas’ orbital evolution.

Lainey et al. (2024) and Rhoden et al. (2024) both modeled the co-evolution of Mimas’ eccentricity and interior, assuming a recent eccentricity-pumping event led to the onset of melting and ocean generation. Both investigations determined that the initial eccentricity was between 2 and 3 times the present value. Over time, tidal dissipation caused both eccentricity decay and thinning of the ice shell, eventually reaching today’s conditions, in which the shell is between 20 and 30 km thick (Tajeddine et al. 2014; Lainey et al. 2024). Based on these results, both studies concluded that Mimas’ ocean has likely emerged within the last ∼10 Myr (Lainey et al. 2024; Rhoden et al. 2024). As described in the following section, a young ocean, in which the ice shell has thinned over Mimas’ recent past, is compatible with Mimas’ geology (Rhoden 2023; Rhoden et al. 2024), although it does not provide as much insight as to Mimas’ initial origin or age.

In a pair of companion papers, Baillié et al. (2019) and Noyelles et al. (2019) investigated a potential history for Mimas in which a recent resonance passage with either Tethys or Enceladus resulted in Mimas migrating inward, increasing its eccentricity. As a result of its migration, Mimas would have opened a gap in Saturn’s rings, perhaps explaining the formation of the Cassini Division (Baillié et al. 2019). The changes in Mimas’ orbit would also lead to enhanced tidal heating within Mimas (Baillié et al. 2019; Noyelles et al. 2019). The scenarios investigated so far either induce too much melting within Mimas to be compatible with its geology or do not reproduce the current orbital elements of the moons (Noyelles et al. 2019). In any case, once melting occurs, and dissipation is further enhanced, the eccentricity drops, eventually causing the moons to exit the resonance (Baillié et al. 2019; Noyelles et al. 2019). At that point, Mimas would return to a state of outward migration, with its eccentricity continuing to decrease (Baillié et al. 2019; Noyelles et al. 2019).

Based on estimated rates of infilling of the Cassini Division, the entire inward and outward migration of Mimas is presumed to have occurred within the past 10 Myr (Baillié et al. 2019; Noyelles et al. 2019). While this proposed history fits with the timescale and sequence of events suggested by Lainey et al. (2024) and Rhoden et al. (2024), the implied eccentricity of Mimas is too high by an order of magnitude, and there is a mismatch in the time since Mimas resumed outward migration (∼1 Myr in Baillié et al. 2019 vs 3–25 Myr in Lainey et al. 2024). It remains to be seen whether these models can be reconciled, or whether Mimas’ emerging ocean is entirely disconnected from the process that opened the Cassini Division.

Modeling of Mimas’ coupled thermal-orbital evolution over billion year timescales has confirmed that, if a primordial Mimas were heated sufficiently to differentiate, it would also lose its eccentricity (Neveu and Rhoden 2017). Any ocean would be lost as a result of eccentricty decay and the subsequent drop in tidal heating, and any geologic features formed in response would need to be obscured by later cratering to match observations. In contrast, a ring-born Mimas could have formed layered (as in Charnoz et al. 2011) while preserving any initial eccentricity and avoiding geologic activity associated with an ocean (Neveu and Rhoden 2019). While the ability to preserve a primordial high eccentricity initially seemed like a valuable attribute of the ring-formation model, the likelihood of a recent eccentricity-pumping event at Mimas means neither model can be ruled out. In this way, the presence of a recently-formed ocean within Mimas creates a disconnect between the present conditions and Mimas’ early origins. The current eccentricity cannot be primordial, and the higher eccentricity at the onset of melting must be a recent event or the entire lifecycle of ocean generation, eccentricity decay, and ocean freezing would have already occurred (Lainey et al. 2024; Rhoden et al. 2024). We do not yet have constraints on Mimas’ eccentricity before the recent eccentricity-pumping event that triggered melting within Mimas.

Reassembly of Mimas as the result of a disruptive impact (e.g., Asphaug and Reufer 2013; Ćuk et al. 2016; Dubinski 2019) could also be compatible with the emergence of an ocean and Mimas’ current high eccentricity, provided that there is sufficient time for Mimas to accumulate craters, including Herschel (see next section). An important open question is whether large impacts, involved in either disrupting precursor moons or generating observed basins on the current moons, would significantly increase Mimas eccentricity (as investigated for Tethys by Zhang and Nimmo 2012) and whether they would be ocean-promoting or ocean-limiting events.

#### Geologic Features and a Young Mimean Ocean

Mimas’ most distinctive geologic feature, the Herschel impact basin, has been used to probe the ice shell thickness at the time of its formation. Numerical modeling of the impact that formed Herschel showed that the ice shell had to be at least 55 km thick in order to match the overall shape and morphology of the basin (Denton and Rhoden 2022). Given that the libration and precession measurements indicate a present-day ice shell no thicker than 30 km (Tajeddine et al. 2014; Lainey et al. 2024), these results support the hypothesis that Mimas’ ice shell has thinned considerably since the formation of Herschel (Denton and Rhoden 2022).

Tidal heating within an ocean-bearing Mimas would generate a globally-averaged surface heat flow of 10s of mW/m^2^ at the present ice shell thickness (Rhoden and Walker 2022). Estimates of historic heat flows for plausible evolutions of a developing ocean and ice shell within Mimas are similar in magnitude (Rhoden et al. 2024). Rhoden et al. (2024) modeled relaxation of a 26-km diameter crater using the highest heat flows identified in their ocean evolution models and determined that the low gravity and cold surface temperatures on Mimas would generate only ∼10 m of topographic change, which is far below the detection limit enabled by *Cassini* image data. Hence, limited crater relaxation is compatible with the development and presence of an ocean.

Mimas lacks the tectonic structures and processes that have been linked to tidal stresses on confirmed ocean worlds, such as Europa and Enceladus (e.g., Greenberg et al. 1998; Rhoden et al. 2010, 2020). Mimas also lacks large canyons, such as those observed on Tethys and Charon, which have been attributed to stresses caused from a past ocean freezing out (Chen and Nimmo 2008; Spencer et al. 2021 and references therein; Rhoden et al. 2023; see also Sect. 5.3), although it has also been suggested that the formation of Ithaca Chasma on Tethys was caused or exacerbated by the Odysseus forming impact (e.g., Moore et al. 2004). When compared with moons for which fracture formation from ocean freezing has been investigated (Nimmo 2004; Rudolph and Manga 2009; Rudolph et al. 2022; Rhoden et al. 2023), cooling cracks on Mimas should be able to transit even thicker ice shells due to Mimas’ lower gravity. In fact, Rhoden et al. (2024) showed that ice shell thickening at Mimas leads to crack penetration to the ocean and surface eruptions of ocean material within all of the evolution models they studied.

The limited tectonic record, and lack of eruptive activity, suggests that Mimas’ ice shell has not thickened enough to form fractures from cooling, and that tidal stresses alone are insufficient to fracture the ice (Rhoden et al. 2017; Rhoden 2023; Rhoden et al. 2024). A recently emerging ocean, and thinning ice shell, are thus compatible with the surface geology of Mimas. In addition, the continued eccentricity decay caused by tidal dissipation within Mimas is likely to cause Mimas to enter a state of ice shell thickening within the next ∼10 Myr, which ought to generate similar tectonic and eruptive activity as we currently observe on Enceladus (Rhoden et al. 2024).

#### Summary of Constraints

Mimas’ geologic record, including the lack of tectonic activity (Rhoden et al. 2017; Schenk et al. 2018), morphology of Herschel (Denton and Rhoden 2022), and limited crater relaxation (Schenk 1989; White and Schenk 2011), is compatible with a young ocean and recently thinning ice shell (e.g., Rhoden et al. 2024). The emergence of an ocean points to an eccentricity-pumping event that triggered melting within the last ∼10 Myr of Mimas’ history (Lainey et al. 2024; Rhoden et al. 2024), although the exact mechanism that raised the eccentricity is still under investigation (e.g., Baillié et al. 2019; Noyelles et al. 2019). Such a recent change in Mimas’ orbit and interior somewhat decouples its present state from its initial formation. In particular, retaining a high eccentricity over long timescales is no longer a strict constraint on Mimas’ evolution as the ocean implies a recent eccentricity-pumping event (Lainey et al. 2024; Rhoden et al. 2024).

Mimas’ differentiated interior (Tajeddine et al. 2014; Noyelles et al. 2019; Caudal 2019; Lainey et al. 2024) requires either global-scale melting (e.g., Neveu and Rhoden 2017), a layered origin such as from ring formation (Charnoz et al. 2011), or some form of impact-induced differentiation (e.g., Barr and Canup 2010). Models that invoke reassembly of Mimas after a disruptive collision or mergers of precursor moons (Asphaug and Reufer 2013; Ćuk et al. 2016; Dubinski 2019) must also provide a pathway to promote or preserve internal layering. Given the ease with which cracks and eruptions form as a result of ocean freezing (Rhoden et al. 2024), any past ocean that formed during differentiation or from an older epoch of high tidal heating, would need to have been lost before the majority of craters were emplaced to erase the evidence. Mimas’ substantial crater population also requires adequate time and impactor sources to develop (e.g., Kirchoff and Schenk 2010; Ferguson et al. 2024) and a surface solid and cold enough to retain crater shapes over the surface age. However, the lower spatial densities of larger craters on Mimas as compared with Tethys and Dione suggests that Mimas is overall younger, and thus, unlikely to be primordial (Ferguson et al. 2024). Finally, Mimas must be able to participate in an eccentricity-pumping resonance within the past ∼10 Myr in order to develop the ocean that exists today (Tajeddine et al. 2014; Lainey et al. 2024; Rhoden et al. 2024).

### Enceladus

#### Overview of Surface Geology

**Fig. 7:**
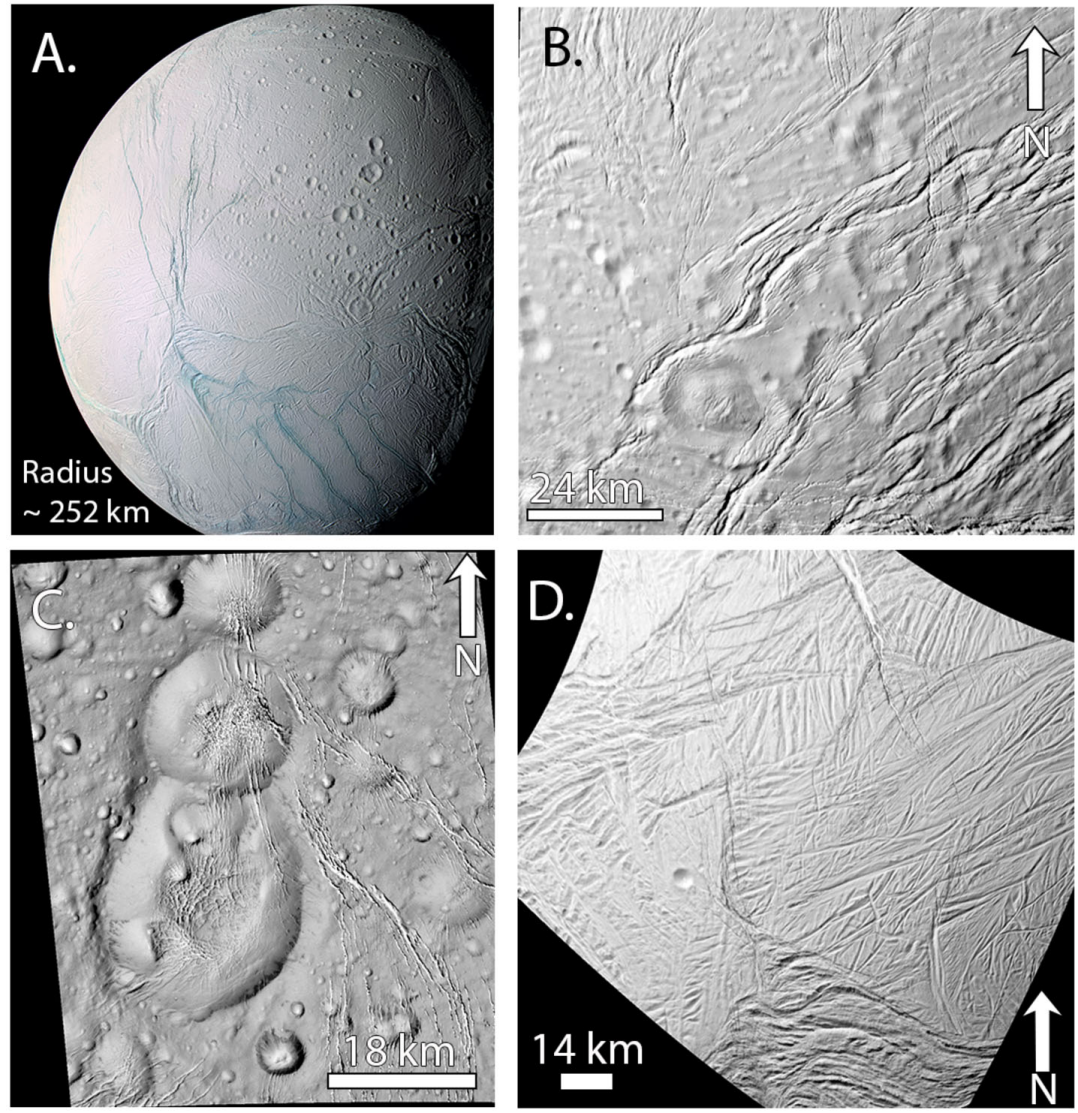
A) View of Enceladus’ south polar terrain, with a focus on the Tiger Stripes fractures. Material from within Enceladus can erupt into space via these fractures. Image PIA07800. B) A transitional region between tectonized and cratered terrains. While craters are present on the surface, they are often overprinted by tectonics and appear to have undergone viscous relaxation. Image N1637465264_1. C) “Snowman” craters near the North Pole of Enceladus. While the cratered terrains in the north are likely representative of older surfaces on Enceladus, these craters are often cross-cut by other fractures. Image N1823513163_1. D) Fractured terrain on Enceladus. Due to the lack of impact craters on this surface, it’s inferred that fracture formation occurred relatively recently in geologic history. Image N1604168315_3

Enceladus’ surface (Fig. 7) is broadly arranged into four terrain types: 1) the cratered terrains co-located with the sub- and anti-Saturn points, 2) the tectonized terrains on the leading and trailing hemispheres, 3) the more heavily cratered, but tectonically-modified, north polar terrain and 4) the crater-free and heavily tectonized south polar terrain (e.g., Crow-Willard and Pappalardo 2015; see also Patterson et al. 2018 and references therein). Despite their appearance, there is evidence that the cratered terrains on the sub- and anti-Saturn points are experiencing recent or current tectonic dissection by tectonic structures called pit chains (Martin et al. 2017; Whitten and Martin 2019; Martin et al. 2023). Pit chains are also found at the boundaries between cratered terrains and tectonized terrains, often in en echelon patterns suggesting shear motions, extending into and across north polar terrains (Martin 2016). Given the heavy focus on the geologically/tectonically active south polar terrain (see below), it is intriguing that terrains along the equator (Martin et al. 2023) and at/near the north pole (Martin 2016) may also be undergoing current (or recent) tectonism.

The south polar terrain and the tectonized leading/trailing terrains are thought to be relatively young, as evidenced by their overall lack of impact craters (Kirchoff and Schenk 2009, 2010). In addition, tectonic terrains of the leading and trailing hemispheres differ in character (Crow-Willard and Pappalardo 2015; Patterson et al. 2018; Patthoff et al. 2022). The leading hemisphere terrain is dominated by ridge and trough structures of varying size and morphology that likely formed under compressional stress (Patthoff et al. 2015). The trailing hemisphere terrain includes two vast regions of tectonically striated plains (Sarandib and Diyar planitia) separated by a complex of ridges called dorsa (Crow-Willard and Pappalardo 2015). The morphology of the dorsa suggests they formed as thrust blocks and implies lithospheric shortening (compressive stress), consistent with the sense of strain inferred for the leading hemisphere (Patthoff et al. 2022). In contrast, the striated plains appear to have formed through extension (Bland et al. 2007), with a deep, graben-like trough (Harran Sulci) separating the tectonic terrains from cratered terrain to the east (Giese et al. 2008). The central portions of the leading and trailing hemispheres are surrounded by complex sets of curvilinear ridges and troughs (e.g., Hamah and Samarkand Sulci) that likely record multiple episodes of tectonism (Crow-Willard and Pappalardo 2015; Patterson et al. 2018). The presence of clear indicators of both extension (the plains and graben) and contraction (the dorsa) is rare on icy moons, and suggests a complex tectonic history in Enceladus’ mid-latitudes.

The south polar terrain (SPT) is characterized by four long, roughly-parallel fissures (dubbed Tiger Stripes) from which emanate the on-going plume activity identified by the *Cassini* mission (Hansen et al. 2006; Dougherty et al. 2006; Porco et al. 2006; Spencer et al. 2006). The volume of plume material emitted from the Tiger Stripes appears to be tidally-modulated (Hurford et al. 2007; Hedman et al. 2013), although it has been challenging to match the variations with tidal-mechanical models (e.g., Běhounková et al. 2017 and references therein). The orientations of the Tiger Stripes, on the other hand, are well-explained by eccentricity-driven tidal stresses in an ocean-bearing Enceladus (e.g., Rhoden et al. 2020), although non-tidal origins for the Tiger Stripes have been proposed (Yin and Pappalardo 2015; Hemingway et al. 2020; Schoenfeld and Yin 2024), which may act independently or in concert with tidal stresses. Variations in Enceladus’ ice shell thickness, as described in the following section, will affect tidal stress magnitudes, perhaps explaining the dichotomy in tidally-driven tectonic activity between the north and south poles (Beuthe 2018; Rhoden et al. 2020). However, the overall magnitudes of tidal stresses are still an order of magnitude lower than the failure strength of ice in laboratory studies (e.g., Collins et al. 2009), suggesting additional stresses are involved in fracture formation.

Stresses due to cooling and freezing of a subsurface ocean are often invoked for moons with tidally-driven tectonics in order to overcome the tensile strength of the ice shell (e.g., Nimmo 2004; Rudolph et al. 2022). In particular, thickening of Enceladus’ ice shell would induce large, isotropic, extensional stresses in the upper portion of the ice shell (Nimmo 2004; Rudolph and Manga 2009; Rudolph et al. 2022), resulting in cracks that propagate radially through shell (Rudolph and Manga 2009; Hemingway et al. 2020; Rudolph et al. 2022). In shells that are thin enough for cooling fractures to transit the entire shell (∼10 km on Enceladus), it takes ∼10 Myr of thickening to connect the surface and the ocean (Rudolph et al. 2022). It seems likely that cooling cracks formed the initiation points of Enceladus’ south polar plumes (Hemingway et al. 2020; Rhoden et al. 2020). However, the extent to which radial cooling cracks are related to the laterally-propagating Tiger Stripe fractures from which the plumes now emanate is not well-understood.

The Tiger Stripes lie atop a region of dense fractures, which includes many crosscutting and overlapping fractures of various orientations and a set of parallel fractures that have been interpreted as paleo Tiger Stripes (Patthoff and Kattenhorn 2011). The history of fracture formation, activation, and decommission at the south pole implies changes in the stress field controlling fracture orientations (Patthoff and Kattenhorn 2011). As discussed more in the following section, non-synchronous rotation (NSR) of Enceladus’ ice shell could plausibly cause the requisite changes in stress over time to explain the orientations of older fractures in the SPT, and the relatively large magnitude of NSR stress may also facilitate fracture formation (Patthoff and Kattenhorn 2011). Whether the past generations of fractures also served as conduits for plumes is presently unknown.

Many craters on Enceladus are heavily relaxed, even near the equator, which is well outside the active south polar region (e.g., Fig. 5). Bland et al. (2012) found that even with 10s of mW/m^2^, applied over millions of years, the measured relaxation could not be reproduced. Only by adding a brief pulse of high heat flow (150 mW/m^2^), along with an insulating regolith that could keep the near-surface temperature higher than the measured value at the surface, could the modeling results begin to approach the levels of relaxation on Enceladus. Muted crater morphologies on Enceladus have been attributed to a combination of viscous relaxation and infilling from plume fallback (Bland et al. 2012). These processes can erase many of the craters with diameters <2 km and >6 km (Kirchoff and Schenk 2009) and help match the observed depth-to-diameter ratios observed on Enceladus. While crater infilling by plume fallback would contribute to muting the short wavelength topography of craters on Enceladus (Bland et al. 2012), thick sequences of plume fallback, on the order of 100s of meters, would take millions or billions of years to deposit across the surface given current plume mass fluxes (Martin et al. 2023).

Composition measurements of material erupting from Enceladus’ plumes indicates on-going hydrothermal alteration, which may be indicative of geologically recent interactions between rock and liquid water (e.g., Hsu et al. 2015). In particular, the detection of H_2_ in Enceladus’ plumes (Waite et al. 2017) suggests that water-rock interactions are on-going. Since rock hydration operates on short timescales (i.e., million years, e.g., Martin and Fyfe 1970), the presence of H_2_ in the plume might indicate that Enceladus’ ocean is young.

#### Interior Structure and Evolution

Enceladus has the highest bulk density of the mid-sized moons (1.6 g/m^3^; Rappaport et al. 2007) suggesting a rock fraction of ∼60% by mass. Enceladus’ shape shows significant deviation from hydrostatic equilibrium in the form of excess flattening, particularly at the south pole (Thomas et al. 2007; Nimmo et al. 2011; Hemingway et al. 2018 and references therein). The shape and gravity data obtained by *Cassini* result in a J_2_/C_22_ of 3.51 ± 0.05 (Iess et al. 2014). When the rapid rotation of Enceladus is accounted for, the J_2_/C_22_ for a hydrostatic Enceladus would be 3.25 rather than 10/3 (Tricarico 2014; McKinnon 2015). Either way, the results suggest that Enceladus’ gravity field is not hydrostatic (Iess et al. 2014; McKinnon 2015). That Enceladus’ shape deviates more from hydrostatic than its gravity implies some amount of compensation of excess topography, which a subsurface ocean could provide.

As discussed in Sect. 4, the lack of hydrostatic equilibrium complicates inferences of Enceladus’ moment of inertia and interior structure. Therefore, many studies have attempted to further constrain Enceladus’ interior from gravity and shape measurements using different approaches to isolate non-hydrostatic components and/or incorporate isostatic compensation (Iess et al. 2014; McKinnon 2015; Čadek et al. 2016; Beuthe et al. 2016; Hemingway and Mittal 2019; Park et al. 2024). These models have sufficient complexity to produce a variety of solutions. However, there is general agreement across these studies that 1) Enceladus is differentiated, 2) the rocky core is ∼190 km in radius and has a low density of 2.3–2.45 g/cm^3^, 3) there is a global ocean that enables compensation of topography, and 4) the mean global ice shell thickness is most likely 20–30 km, with a total hydrospheric thickness of ∼60 km. Enceladus’ low core density may be the result of high porosity, with ice or brines in the pores (Roberts 2015; Choblet et al. 2017), although the porosity of the core is not well-constrained. Assuming uniform porosity, a grain density of 3.1 g/cm^3^ and a brine density of 1.03 g/cm^3^, the average porosity of the core would be ∼35%.

Enceladus has a large physical libration; the original study using *Cassini* imagery found a libration amplitude of 0.12° ± 0.014° ($2\sigma $) that was 180° out of phase with the eccentricity libration (Thomas et al. 2016). The libration amplitude was revised down in a later analysis to 0.091° ± 0.009° ($3\sigma $; Park et al. 2024). With either value, the large libration amplitude requires a global ocean to mechanically decouple the ice shell from the interior (Thomas et al. 2016; van Hoolst et al. 2016; Park et al. 2024), consistent with the inferences from the gravity studies (e.g., Hemingway and Mittal 2019). However, the compensation depth of the ice shell (i.e., its thickness) depends on the assumed libration amplitude. Hence, using the value from Thomas et al. (2016), the ice shell would have a global average thickness of 21–26 km if it behaves entirely elastically or 14–19 km if it is viscoelastic. Park et al. (2024) find a global average ice shell thickness of 27 to 33 km based on their libration analysis.

Enceladus’ ice shell varies in thickness with latitude, with the south pole being the thinnest, then the north pole, and then equatorial regions (Iess et al. 2014; McKinnon 2015; Beuthe et al. 2016; Thomas et al. 2016; Čadek et al. 2016, 2019). Because the estimates are model-dependent, there is disagreement as to just how thick the shell currently is at the south pole, from less than 5 km to at least 10 km. Hypotheses for the source of the thickness variations include, but are not limited to, stochastic effects of interior evolution leading to one hemisphere experiencing more heating/melting than the other, patterns of convection (e.g., Běhounková et al. 2012 and references therein), or a giant impact that led to reorientation (Peale and Greenberg 2007; Roberts and Stickle 2021). In addition, gravity data suggests that the ice shell has thickened with time (Čadek et al. 2019), which could have facilitated formation of the conduits that enable Enceladus’ plumes (Hemingway et al. 2020; Rudolph et al. 2022).

The evolution of Enceladus’ interior may provide an explanation for the changes in fracture orientations at the south pole and the current locations of tectonized terrains near the equator (Rhoden et al. 2020). If Enceladus were frozen, but separated into an ice layer over a rocky interior, the pattern of tidal heating would be nearly inverted from the pattern with an ocean (e.g., Roberts 2015). In that case, tidal heating would be concentrated at the equator, centered on the leading and trailing points. These are the locations at which we observe older tectonized terrains (Crow-Willard and Pappalardo 2015). If Enceladus then transitioned from a frozen world to one with a subsurface ocean, these locations would have experienced tidal stress that may have led to fracturing. Once the ocean became global in extent, the pattern of tidal heating and stress would change such that maximum tidal heating and stress were generated at the poles (Roberts 2015; Rhoden et al. 2020). If the Tiger Stripes, which are the youngest geologic features at the south pole (Crow-Willard and Pappalardo 2015; Patterson et al. 2018), formed as a result of ice shell thickening (as in Hemingway et al. 2020; Rudolph et al. 2022), it would imply that Enceladus has since transitioned from a growing ocean to a shrinking one, which is consistent with the gravity measurements (Čadek et al. 2019).

Because the subsurface ocean decouples Enceladus’ ice shell from its interior, the shell can spin via a process called non-synchronous rotation (NSR). As a result of the ice shell’s motion, regions would move to new locations, relative to the direction of Saturn, thus exposing them to a different tidal stress field. In addition, NSR can impart large stresses on the shell that can facilitate fracture formation (e.g., Kattenhorn and Hurford 2009), although the relative importance of NSR versus diurnal tidal stress at Enceladus is not yet known. Slow NSR has been suggested from an analysis of fracture orientations at Enceladus’ south pole (Patthoff and Kattenhorn 2011) and the systematic rotation of pit chain orientations within Enceladus’ cratered terrains (e.g. Martin et al. 2017, 2023). If NSR has been acting to reorient the ice shell in longitude, it would imply that the tectonized terrains near the equator, which are currently aligned with the sub- and anti-Saturn points, have also moved since they formed and are at their current locations by happenstance. In addition, a minimum of millions of years would be required to create the observed fracture population within the south polar region, depending on the rotation period (Patthoff and Kattenhorn 2011), which limits the youth of tectonic activity on Enceladus overall.

*Cassini* measurements revealed that Enceladus is producing a tremendous amount of heat, particularly in the south polar region (Spencer et al. 2006; Howett et al. 2011), and high heat flows are recorded in heavily relaxed craters even well outside the SPT (Bland et al. 2012). Although tidal heating can be produced in Enceladus’ ice shell (e.g., Soucek et al. 2019), and to lesser extent in its ocean (Hay and Matsuyama 2019; Rovira-Navarro et al. 2019), such high heat flows may require tidal dissipation in the core of Enceladus. Several studies have shown that a porous core within Enceladus would generate a significant amount of tidal dissipation (e.g., Roberts 2015; Choblet et al. 2017; Rovira-Navarro et al. 2022). The link between tidal dissipation, core characteristics, and formation is discussed more in Sect. 5.6.

Enceladus maintains a mean motion resonance with Dione (e.g., Meyer and Wisdom 2008; Ćuk and El Moutamid 2022), which enables its eccentricity to remain relatively high (0.0047) in spite of its extreme dissipation. Whether Enceladus is currently in a state of equilibrium tidal heating or oscillating around an equilibrium depends on how dissipation is partitioned between Enceladus and Dione, as well as the evolution of Saturn’s Q, as discussed in detail by Nimmo et al. (2023). In addition, Enceladus may have experienced past epochs of higher eccentricity during the complex dynamical evolution of all five mid-sized moons (e.g., Meyer and Wisdom 2008; Běhounková et al. 2012; Neveu and Rhoden 2019; Noyelles et al. 2019; Nakajima et al. 2019; Ćuk and El Moutamid 2022). For a more complete review of dissipation within Enceladus, see Nimmo et al. (2023).

#### Summary of Geologic Constraints

Models of Enceladus’ formation and evolution need to account for the following observations. First, Enceladus displays differences in geologic activity that vary both spatially and temporally (Crow-Willard and Pappalardo 2015; Patterson et al. 2018). Tectonic features at the south pole have been linked to tidal stress and stresses from a freezing ocean (e.g., Beuthe 2018; Hemingway et al. 2020; Rhoden et al. 2020; cf., Yin and Pappalardo 2015), although stress from non-synchronous rotation may have also played a role (Patthoff and Kattenhorn 2011). The origins of older fractures both within the south polar terrain and in equatorial regions are less certain (e.g., Patterson et al. 2018). One potential explanation for Enceladus’ tectonic history is the development of an ocean that has since entered an epoch of freezing (Rhoden et al. 2020). That we observe the most tectonic activity where the ice shell is thinnest is not surprising given that tidal stresses are enhanced at locally thin regions (e.g., Beuthe 2018), although the process(es) that led to the south pole being thin relative to the global average is still debated (e.g., Běhounková et al. 2012; Roberts and Stickle 2021). Overall, the evolution of Enceladus’ interior, orbit, and rotation state must account for the observed geology and how features have changed with time.

Second, the present-day heat flux is quite high, particularly at the south pole (Spencer et al. 2006; Howett et al. 2011), and craters even well outside the south polar terrain imply high heat flows (Bland et al. 2012). These results require high dissipation in the present as well as in the past (see reviews by Nimmo et al. 2018, 2023). Enhanced tidal dissipation within a porous (also called “fluffy”) core may be critical to explaining such high heat flows (Roberts 2015; Choblet et al. 2017; Rovira-Navarro et al. 2022). Hence, both the assembly and thermal evolution of Enceladus must be compatible with a core that has significant pore space within the rock, to generate the observed dissipation while matching the low core density.

### Tethys

#### Overview of Surface Geology

**Fig. 8:**
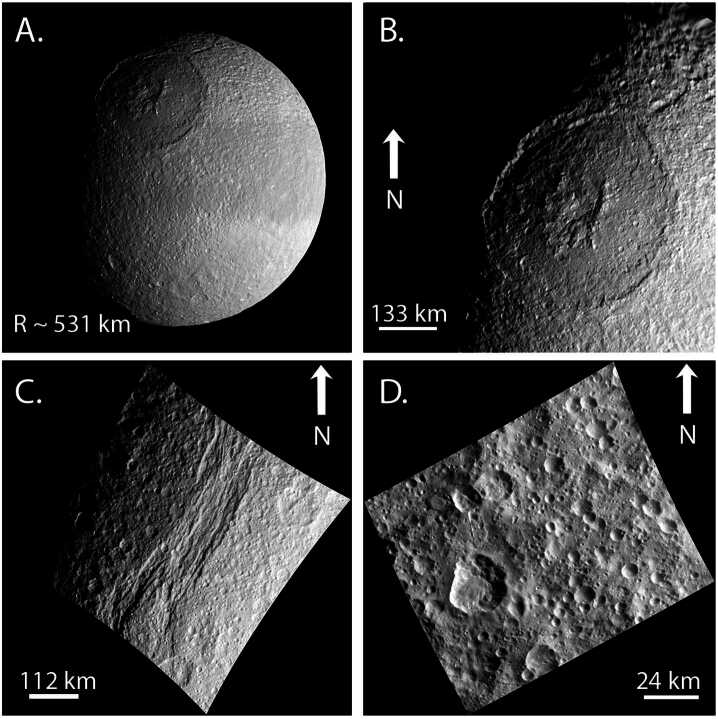
A) Full disk image of the leading hemisphere of Tethys, with a focus on the Odysseus impact basin (D = 445 km). Image PIA08400. B) Closer view of the Odysseus basin and its cratered interior in which the central pit of the basin is clearly visible. Image N1567098978_1. C) Portion of the Ithaca Chasma canyon system, which stretches ∼1800 km across the surface of Tethys. Image N1489061272_1. D) Close up of cratered terrain on Tethys, including some instances of mass wasting. Image N1713137226_1

Tethys’ surface is heavily cratered (Fig. 8), including the large (D = 445 km) impact basin, Odysseus, which displays a central depression along with interior mounds and other topographic features (Schenk et al. 2018). Crater counts within Odysseus suggest that it is ∼4–9 times younger than the heavily cratered terrains on Tethys (Kirchoff and Schenk 2010). Crater SFDs for the most heavily cratered regions of Tethys are similar to those on Dione and Mimas, but Tethys shows less variation in the slope of the crater SFD (i.e., flatter R-values) particularly between roughly 6–20 km in diameter, lower crater density from roughly 7 to 100 km diameters (i.e., lower R-values), and the strong dip in crater density occurs at D ∼80–110 km rather than at D ∼100–120 km as observed on Dione (Kirchoff and Schenk 2010; Kirchoff et al. 2018); these trends are apparent in Fig. 2.

While there are some variations in crater density across different regions of Tethys, there are no regions in which cratering is sparse (Kirchoff and Schenk 2010; Kirchoff et al. 2018; Ferguson et al. 2020). For example, there is a ∼600 km wide region on Tethys’ leading hemisphere that displays a dearth of craters with D > 50 km but is still densely cratered at smaller sizes (Ferguson et al. 2020) and is relatively smooth as compared with the very rugged terrain that is observed across most of Tethys (Moore and Ahern 1983; Schenk et al. 2018). Whether this region was affected by the formation of Odysseus (e.g., Moore et al. 2004; Bruesch and Asphaug 2004), which is close to antipodal, or by an ancient cryovolcanic flow (Moore and Ahern 1983), is still undetermined. There are no other features thus far associated with cryovolcanism on Tethys, and a search for plume activity at Tethys with the *Cassini* Ultraviolet Imaging Spectrograph and Visible and Infrared Mapping Spectrometer yielded negative results (Buratti et al. 2018).

Based on regional crater maps of Tethys, the Case B production function (Zahnle et al. 2003; see also Sect. 3.1) deviates more from the SFDs of craters larger than D = 4–8 km (Ferguson et al. 2020) than at Dione (Ferguson et al. 2022a) or Mimas (Ferguson et al. 2024). For smaller craters, Case A provides a better fit to the data than Case B in most regions, but it then vastly overpredicts the number of craters with D > 4 km (Ferguson et al. 2020). The larger mismatch between Case B and craters on Tethys suggests differences between the planetocentric impactor population at Tethys as compared with Dione and Mimas (e.g., Ferguson et al. 2024).

As discussed in Sect. 3.3, Tethys also has a substantial population of elliptical craters, the majority of which lie within 30° of the equator and are oriented with their long axes pointed east-west (Ferguson et al. 2022b). There is a smaller population of elliptical craters with isotropic orientations that are more globally distributed. Elliptical craters are more likely to form in slow, oblique impacts (e.g., Collins et al. 2011; Elbeshausen et al. 2013), suggesting a planetocentric origin due to their significantly lower impact speeds (Alvarellos et al. 2017). Polygonal craters have also been identified on Tethys (Ferguson et al. 2020); this crater morphology is thought to indicate interactions with fractures, which may not be visible at the surface (Beddingfield et al. 2016).

Tethys displays a global-scale (∼1800 km) rift zone called Ithaca Chasma, which roughly follows a great circle that runs through the sub-Saturn point (Moore and Ahern 1983; Moore et al. 2004). There are several hypotheses for the formation of Ithaca Chasma, including a response to the Odysseus-forming impact (Moore et al. 2004), tidal stresses from orbital recession after an eccentricity-pumping resonance passage (e.g., Hussmann et al. 2019), and at least partial freezing of a subsurface ocean (e.g., Chen and Nimmo 2008). One reason the impact origin has been strongly considered is the apparent geographical correlation between Odysseus and Ithaca Chasma. Specifically, the canyon system roughly follows a great circle that is concentric to Odysseus (Smith et al. 1981; Schenk et al. 2018). Updated crater counts suggest that Ithaca Chasma predates Odysseus, which is the main argument against the impact origin, but there is enough uncertainty in the ages that this hypothesis cannot be fully ruled out (Kirchoff and Schenk 2010; Stephan et al. 2016; Ferguson et al. 2020, 2022b, 2022c).

Complicating the origin of Ithaca Chasma is the source of stress that fractured the ice shell. One option is orbital recession of Tethys, which can lead to a collapse of the tidal bulge, generating large stresses (e.g., Hussmann et al. 2019). However, the orientation of Ithaca Chasma is inconsistent with that stress pattern, leading Hussmann et al. (2019) to suggest that additional stresses were involved in the canyon’s formation. Other sources of tidal stresses have not been fully vetted against the characteristics of Ithaca Chasma. Tidal stresses from eccentricity or obliquity, alone, may be too small to overcome the tensile strength of ice (see discussion in Collins et al. 2009, for example); tidal stresses induced from rotations of the ice shell (e.g., non-synchronous rotation or polar wander) can be much larger, but these stresses have not yet been investigated as sources for Ithaca Chasma’s formation. It is also possible that freezing of a subsurface ocean facilitated fracture formation, as it induces large tensile stresses in the ice shell (e.g., Nimmo 2004) and may act in concert with other stress sources to generate tectonic patterns (as suggested by Hussmann et al. 2019). Models that invoke tidal stress from orbital recession and/or stresses from a freezing ocean also require passage through a mean motion resonance that pumped Tethys’ eccentricity (e.g., Chen and Nimmo 2008; Hussmann et al. 2019).

In addition to Ithaca Chasma, Tethys displays somewhat sparse, globally-distributed tectonic features, generally in the form of troughs, grooves, or pit chains (Moore and Ahern 1983; Schenk et al. 2018; Ferguson et al. 2020). In general, Tethys’ tectonic features have been interpreted as extensional in origin, although most features are not well-explained by any particular formation model (e.g., Schenk et al. 2018). One region near Odysseus, in which higher-resolution *Cassini* imagery allowed for detailed mapping, showed a higher density of grooves and significantly more small craters (D = 1–4 km) than were identified in similar image data sets in other regions (Ferguson et al. 2020), perhaps suggesting a causal link between impacts and grooves in the region.

Craters on Tethys record variable heat flows, ranging from a few mW/m^2^ to ∼100 mW/m^2^ (White et al. 2017), which suggests either spatial or temporal changes in heat flow. Shallow angles of normal faults also indicate extensive viscous relaxation of the fault scarps, implying high heat flows (Beddingfield et al. 2015). Analysis of the flexural uplift of the margin of the Ithaca Chasma tectonic structure (Fig. 8) indicates 18–30 mW/m^2^ along the northern limb of the canyon system (Giese et al. 2007) and 12–39 mW/m^2^ across the south limb (Beddingfield et al. 2023), suggesting that large regions of the satellite experienced similar heat fluxes. However, measurements for Ithaca Chasma’s heat flux are lower than what is expected for the nearby Telemus basin (Beddingfield et al. 2023). Ithaca Chasma is thought to be older than other regions of Tethys (e.g., Giese et al. 2007), whereas relaxed craters may have a variety of ages, so one interpretation of the heat flow results is that Tethys has undergone multiple epochs of high heat flow (Beddingfield et al. 2023).

#### Interior Structure and Evolution

*Cassini* measurements provided few constraints on the present-day interior structure of Tethys. The bulk density of 0.984 g/cm^3^ limits the amount of rock present, even if there is substantial porosity in Tethys’ outer layer. The rock fraction is about 7 wt.%, although we do not yet know whether the rock is globally distributed or concentrated in a core (Castillo-Rogez et al. 2018, and references therein).

Given Tethys’ low rock fraction and relatively small size (r = 531 km), it is unlikely that radiogenic or primordial accretionary heating contributed to the high heat flows preserved in Tethys’ geologic record, leaving tidal heating as the most likely candidate (e.g., Castillo-Rogez et al. 2018; Tian and Nimmo 2020). Presently, tidal effects from orbital eccentricity are negligible due to Tethys’ nearly circular orbit. However, the eccentricity may have been higher in the past (see below). In addition, if Tethys’ spin pole is tilted, which may result from its inclined orbit, obliquity tides may be able to generate substantial tidal heating and stress (e.g., Rhoden et al. 2010; Chen et al. 2014). The extent of tidal heating will depend on whether the interior of Tethys is readily deformable. For example, tidal heating from obliquity-raised tides in the ocean, which could be significant on Tethys (Chen et al. 2014), would not be available if Tethys has remained frozen.

Past tidal heating within Tethys has been inferred from the moon’s global shape, under the simplifying assumption that ice shell thickness variations mimic the tidal heating pattern (Gyalay and Nimmo 2023). The best fits to the global shape support the idea that obliquity tides have played a key role in shaping Tethys. However, tidal heating in the best fit model would produce less than 2 mW/m^2^, which is far less heat than is inferred from the other geology (e.g., White et al. 2017), and less than would be required to create the differentiated (but presently frozen) interior structure used in the fits. Therefore, the model still relies upon a past high eccentricity that generated high heat flows, with the shape emplaced later, after the orbit circularized but the obliquity was high (Gyalay and Nimmo 2023).

Thermal-orbital evolution models of the five mid-sized moons, for a variety of assumptions about Saturn’s Q, revealed that Tethys’ history of mean motion resonance passages could have generated an ocean that persists to the present day (Neveu and Rhoden 2019). Whether the simulations assume a primordial origin in the CPD or a late, layered origin within the rings, the ocean within Tethys could have been 100s of km thick in the past, with a much thicker ice shell today (Neveu and Rhoden 2019). The models also reproduce Tethys’ nearly circular present-day orbit. Circularization results from tidal dissipation caused by an initially high eccentricity, and the decrease in eccentricity leads to rapid thickening of the ice shell (Neveu and Rhoden 2019). Obliquity was not included in the model. Additional studies of past mean motions resonances between Tethys and other Saturnian moons (e.g., Chen and Nimmo 2008; Hussmann et al. 2019) can also produce enhanced tidal dissipation that could produce the high heat flows inferred from Tethys’ geologic features (e.g., Giese et al. 2007; Beddingfield et al. 2015; White et al. 2017), although ocean evolution within Tethys was not tracked. More work is needed to quantify the combinations of eccentricity, obliquity, and interior structure that can produce 10s of mW/m^2^ of heat within Tethys.

Partial or complete freezing of an interior ocean is a predicted outcome of the thermal-orbital evolution models (Neveu and Rhoden 2019) and generates extensional stresses in the upper half of the overlying ice shell (e.g., Nimmo 2004). Within the Saturn system, radially-propagating fractures that form as a result of ocean freezing have been modeled for Enceladus and Mimas (Rudolph et al. 2022; Rhoden et al. 2024). Tethys’ surface gravity is larger than that on either moon, so we can infer that fractures formed from ocean freezing will be less able to fully penetrate ice shells of comparable thickness (e.g., ∼10 km on Enceladus and up to ∼50 km on Mimas; Rudolph et al. 2022; Rhoden et al. 2024). The limited evidence of flows or eruptions on Tethys suggests that the hypothesized ocean remained thin enough that fractures could not fully penetrate the thick ice shell and tap the ocean. However, if the smooth region on Tethys leading hemisphere is shown to be the result of cryovolcanic flows (e.g., Moore and Ahern 1983), it may argue for at least one fully penetrating fracture in Tethys’ history. A study of the conditions under which freezing of a subsurface ocean within Tethys could have formed deep fractures, enabling the formation of Ithaca Chasma, while avoiding widespread eruptions, may help constrain the range of thermal histories that can account for Tethys’ geology and the inferred heat flows through time.

Impact formation models have shown that crater size and morphology are sensitive to the structure and thermal profile of an ice shell, potentially even revealing the presence of a subsurface ocean (e.g.,Turtle and Pierazzo 2001; Senft and Stewart 2011; Bray et al. 2014; Silber and Johnson 2017; Denton et al. 2021; Denton and Rhoden 2022; Bjonnes et al. 2022). A detailed study of the Odysseus-forming impact, with varying ice shell thicknesses (including a frozen Tethys) may provide additional constraints on the timing of the impact with respect to the evolution of Tethys’ interior and orbit. Moreover, the formation and subsequent relaxation of Odysseus may help constrain whether and when Tethys had a subsurface ocean and at what depth, as was recently done for Herschel on Mimas (Denton and Rhoden 2022).

#### Summary of Geologic Constraints

Hypotheses regarding the formation and evolution of Tethys need to satisfy the following geologic constraints. First, as with Mimas, there must be adequate time and impactors to generate Tethys’ considerable cratering record, including formation of the Odysseus impact basin. The age of Odysseus is not well-constrained, in part due to the uncertainties in impactor source populations described in Sect. 3; estimates range from 200 Myr to 3.9 Gyr (Giese et al. 2007; Dones et al. 2009; Kirchoff and Schenk 2010; Kirchoff et al. 2018). Tethys also has a substantial elliptical crater population, with a curious spatial distribution that requires an impactor source that is not heliocentric (see Sect. 3; Ferguson et al. 2022b). Hence, Tethys’ bombardment history must account for the formation and dispersal of this additional population of planetocentric impactors.

Second, Tethys must have experienced at least one epoch of high heat flow, which can most easily be produced by tidal heating after an eccentricity-pumping resonance passage, in order to account for the extensive crater relaxation (White et al. 2017) and the heat flow implied by the topography of Ithaca Chasma (Giese et al. 2007; Beddingfield et al. 2015). However, the interior structure of Tethys during that time period will have a major control on the extent of tidal dissipation and heating. Coupled thermal-orbital evolution models suggest that Tethys could have a long-lived subsurface ocean (e.g., Neveu and Rhoden 2019), which may provide a heat source for crater relaxation via eccentricity and/or obliquity tidal heating (e.g., Chen et al. 2014), although the link between tidal heating and Tethys’ varying states of crater relaxation has not been fully investigated. Whether these heat sources are simultaneously compatible with Tethys’ global shape (e.g., Gyalay and Nimmo 2023) and other geologic features is still unknown.

Third, a mechanism is needed for the formation of Ithaca Chasma that can satisfy the following constraints: the elastic thickness of the ice shell was 5–7 km at the time of formation (Giese et al. 2007), there was a source of stress high enough to induce tensile failure in the ice such as freezing of a subsurface ocean (e.g., Chen and Nimmo 2008), and there was a heat source sufficient to explain the associated relaxation and shallow fault angles (Giese et al. 2007; Beddingfield et al. 2015). A mechanism that can also explain the location and orientation of Ithaca Chasma would be particularly compelling (e.g., discussion in Schenk et al. 2018).

Finally, Tethys has a substantially lower density than the other mid-sized moons, particularly its neighbors, Enceladus (interior) and Dione (exterior). None of the current formation models provide a compelling explanation for Tethys’ very low density; CPD formation would imply a density gradient that is not observed (e.g., Canup and Ward 2006, 2009; Mosqueira et al. 2010), whereas ring-based formation (e.g., Charnoz et al. 2011) and early mergers (Asphaug and Reufer 2013) appeal to stochastic effects to reproduce the variable rock contents of the moons. Reassembly of a precursor moon would imply that either more rock than ice was lost in the collision or that the low rock fraction predated the collision, which would still require explanation. In the former case, Tethys’ density could provide a key constraint on the conditions of the impact that led to its final assembly.

### Dione

#### Overview of Surface Geology

**Fig. 9:**
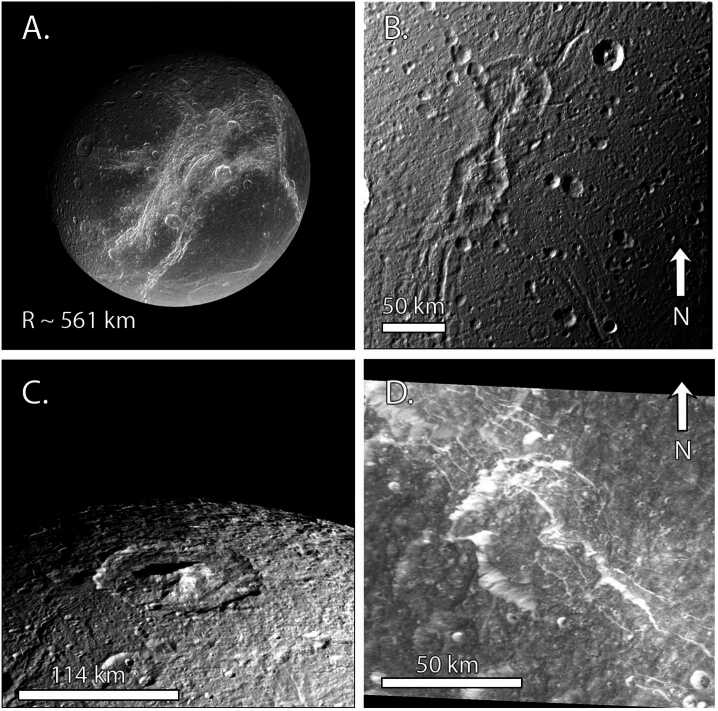
A) Full disc view of Dione’s trailing hemisphere showcasing the wispy terrain (PIA08526). B) Cratered terrain on Dione’s leading hemisphere. Pictured off-center are the heavily degraded and modified craters of Murranus (56.8 km) and Metiscus (43.8 km). Image N1820417749_1. C) Oblique view of Erulus crater (120 km), which is thought to be heavily relaxed (White et al. 2017), with a focus on the central peak complex. Image N1665975031_1. D) Amastrus crater (62.4 km) located within the wispy terrain on Dione’s trailing hemisphere, illustrating the combined effects of cratering and tectonic resurfacing on Dione. Image N1507743880_2

After Enceladus, Dione is the most heavily tectonized of the mid-sized moons (Fig. 9; Schenk et al. 2018 and references therein). The majority of Dione’s tectonism is preserved in the heavily dissected “wispy” terrains concentrated within the trailing hemisphere (Wagner et al. 2006; Moore and Schenk 2007). With few overprinted impact craters, the fractures within the wispy terrains are likely some of the most recent geologic structures preserved on Dione’s surface (Kirchoff and Schenk 2015). Older tectonic activity on Dione includes wide troughs that may be graben and at least one large ridge, Janiculum Dorsa, on the leading hemisphere (Hammond et al. 2013). Inferences of early tectonism are supported by numerous polygonal craters found across terrains on Dione, both in and outside the wispy terrain (Beddingfield et al. 2016), implying that widespread fracturing has been occurring throughout Dione’s history. The relative ages of these periods of tectonism are supported by crater densities, where the terrains around Janiculum have a greater crater density than those within the wispy terrains (Kirchoff and Schenk 2015).

Resurfacing obscures older tectonic features, making it difficult to determine their original distribution and, thus, test likely dominant stress mechanisms. However, the graben and normal fault scarps within the wispy terrains show good matches with diurnal tidal and non-synchronous rotation stress fields (Collins et al. 2010; Martin et al. 2016). The magnitude of stresses from either of these sources would be amplified in the presence of a global subsurface ocean. In fact, an ocean is necessary to enable NSR by mechanically decoupling the ice shell from the interior (e.g., Helfenstein and Parmentier 1985). Although this first order result requires further analysis, the distribution and orientation of fractures within Dione’s wispy terrains suggest a global ocean at some point in Dione’s evolution. In total, the extensional features on Dione are consistent with ∼1% surface expansion (Collins et al. 2010), such as from partial freezing of a subsurface ocean, although fracture formation from ocean freezing has not been explicitly modeled for Dione.

In addition to Dione’s extensive tectonic activity, evidence for cryovolcanism is preserved in the geologic record. Smooth plains that dominate the trailing hemisphere have been suggested to have formed by some amount of cryovolcanic resurfacing (e.g. Smith et al. 1981; Plescia and Boyce 1982; Plescia 1983; Moore 1984; Morrison et al. 1984). Furthermore, centered within the smooth terrains are Murranus and Metiscus craters within Fidena Fossae (Fig. 9B). The morphology of these craters is not indicative of impact craters, and they are hypothesized to be cryovolcanic vents (Schenk and Moore 2009; Kirchoff and Schenk 2015). It is possible that viscous relaxation also played a role in forming the smooth terrains (Hammond et al. 2013). Overall, more work is needed to further understand the endogenic geologic processes that have shaped the surface of Dione.

The *Cassini* mission revealed plasma streams originating separately from Dione and Tethys (Burch et al. 2007; Buratti et al. 2011), as well as the possibility of a tenuous atmosphere around Dione (Clark et al. 2008). These results suggest some amount of on-going endogenic activity, which could be cryovolcanic. Despite detailed searches for active plumes over the course of the *Cassini* mission (Buratti et al. 2011, 2018), none were detected at Dione. The possibility remains that Dione is still geologically active, but future mission work will be required to solve this mystery.

**Fig. 10:**
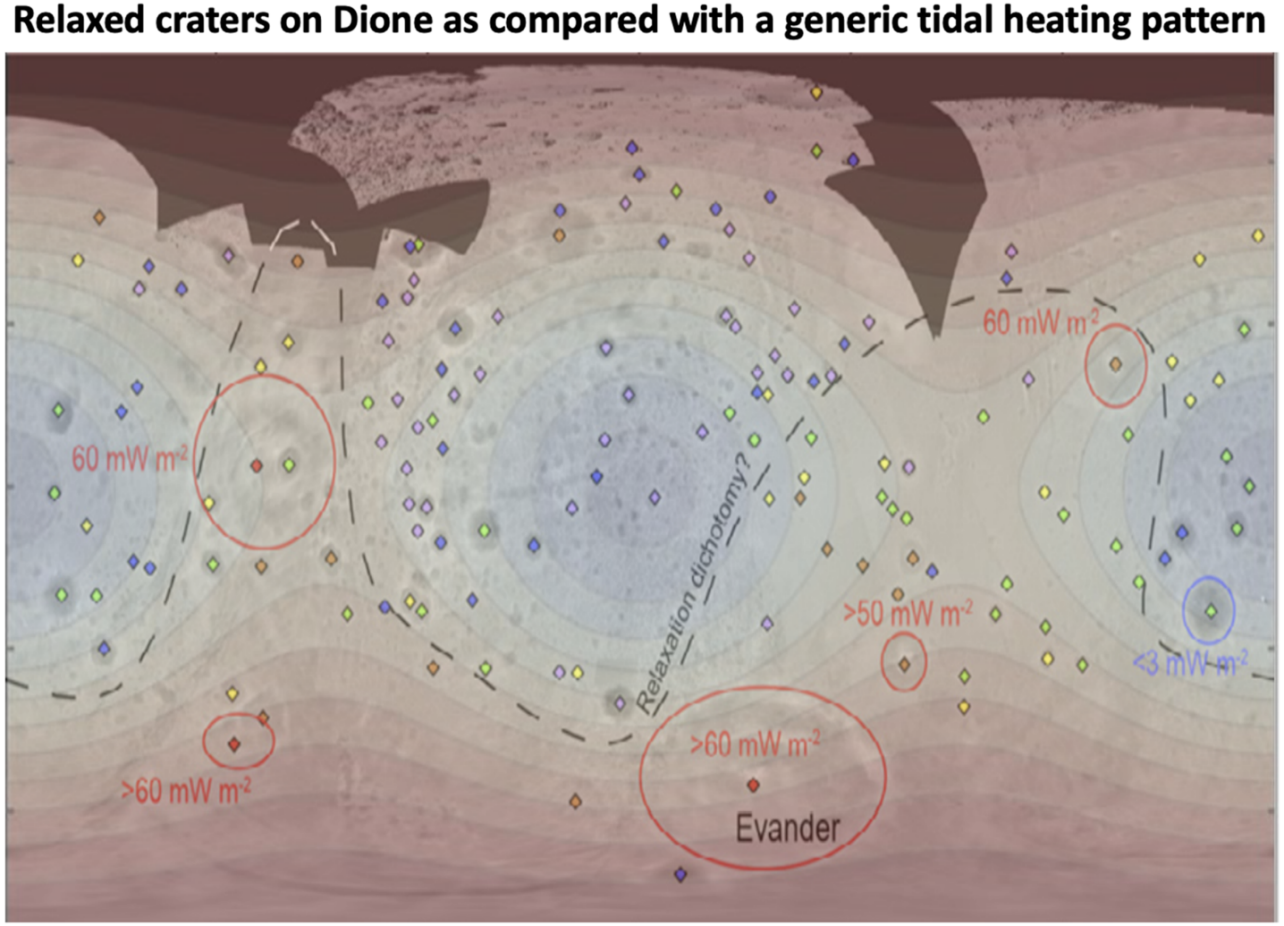
Dione’s craters record variable rates of relaxation, which could mean they are different ages or that the heat flow that led to relaxation was spatially-variable. Here, we overlay the relaxation results from White et al. (2017) on a generic contour map of eccentricity-driven tidal heating (similar to the one shown in Roberts 2015). Warm colors represent higher heat flows relative to the cooler colors. There is good correlation between areas of higher relaxation (and inferred heat flow) and the regions of higher tidal heating, but the fit is not perfect. Generating these high heat flows from tides alone is challenging without both a subsurface ocean and a higher past eccentricity, owing to Dione’s distance from Saturn. It is also possible that the initial depths assumed for the craters on Dione are too large, leading to an overestimate of the past heat flow (e.g., Sect. 3.4)

Dione’s long-wavelength topography (Nimmo et al. 2011), along with the topography of Janiculum Dorsa (Hammond et al. 2013), the shallow dip angles of normal faults (Beddingfield et al. 2015), and Dione’s relaxed craters (White et al. 2017) all indicate a history of high heat flows. Craters record variable heat flows from only a few mW/m^2^ to ∼100 mW/m^2^. The large Evander basin is heavily relaxed, with models suggesting heat flows over 60 mW/m^2^ (White et al. 2017). A rough geographical boundary can be drawn between groups of highly relaxed and minimally relaxed craters (White et al. 2017). As shown in Fig. 10, the pattern correlates with the spatial variations in tidal heating from eccentricity, although the present-day eccentricity (0.0022), along with Dione’s large orbital distance, is unlikely to generate high heat flows at present. Furthermore, there are only a handful of highly relaxed craters on Dione, so the pattern may not be significant (White et al. 2017).

#### Interior Structure and Evolution

Dione’s average density is about 1.478 g/cm^3^, which corresponds to an ice fraction of about 48 wt.% or 75 vol.% assuming no porosity (Castillo-Rogez et al. 2018). Gravity data from multiple *Cassini* flybys indicate a J_2_/C_22_ of 4.102 ± 0.044, which is far from the hydrostatic value (Zannoni et al. 2020). Dione’s shape also deviates from hydrostatic, with a −H_20_/H_22_ of 4.9 ± 0.4 (Zannoni et al. 2020). The fact that the shape deviates more than the gravity suggests partial compensation of excess topography (e.g., Hemingway et al. 2016, 2018; Zannoni et al. 2020). Studies combining gravity, shape, compensation models, and plausible interior structures suggest a differentiated interior with a present-day ocean, likely buried under ∼100 km of ice, which provides some compensation of topography (Beuthe et al. 2016; Hemingway et al. 2016; Zannoni et al. 2020). However, more data is needed before this interpretation can be considered robust (e.g., Castillo-Rogez et al. 2018; Zannoni et al. 2020).

Thermal-orbital evolution models do not predict a present or recent ocean within Dione (Neveu and Rhoden 2019). Formation from the rings seems to generate a longer-lived past ocean than primordial formation (e.g., Neveu and Rhoden 2019). It is possible that the models have neglected an important source of heat, such as core dissipation, an additional unidentified resonance passage, or fluctuations in heating caused by a change in dissipation within Saturn.

Despite the challenges of producing an ocean within Dione, interpretations of its geology generally rely upon an ocean’s existence to enhance stresses and/or heat flows (Collins et al. 2010; Martin et al. 2016; White et al. 2017). For example, a past epoch of higher eccentricity has been invoked to explain the high heat flows recorded by relaxation of Dione’s craters (e.g., White et al. 2017). In addition, Dione displays globally-distributed extensional tectonic features attributed to ocean freezing (Collins et al. 2010), while also showing evidence of cryovolcanic flows (e.g. Smith et al. 1981; Plescia and Boyce 1982; Plescia 1983; Moore 1984; Morrison et al. 1984). Ocean freezing has also been suggested as a key mechanism in creating Enceladus’ south polar tectonic features and enabling eruptions (Hemingway et al. 2020; Rudolph et al. 2022) and for facilitating the creation of the Ithaca Chasma canyon system on Tethys (Moore and Ahern 1983; Schenk et al. 2018). These observations lead to the question of why the same geologic process (i.e., ocean freezing) manifests so differently across the moons.

Because Dione’s surface gravity is higher than Enceladus and Tethys, the propagation depth of cooling cracks will be limited in comparison (e.g., Rudolph and Manga 2009; Rudolph et al. 2022), perhaps affecting the resulting tectonic features. In addition, if Dione has always had a relatively thin ocean, buried beneath ∼100 km of ice (e.g., Zannoni et al. 2020), it is possible that minimal ocean freezing has occurred, limiting the overall surface expression. In that case, however, the smooth plains and cryovolcanic features on Dione could not be the result of cooling cracks and ocean overpressure driving eruptions (cf., Rudolph et al. 2022).

#### Summary of Geologic Constraints

Models of the formation and evolution of Dione must provide mechanisms to produce a long history of geologic activity – including multiple epochs of tectonic activity and cryovolcanism – and a heat source that can account for relaxation recorded in surface features (e.g., Hammond et al. 2013; White et al. 2017) and in Dione’s global shape (Nimmo et al. 2011). Dione’s inferred high heat flows (White et al. 2017), which are suggestive of enhanced tidal heating in an ocean-bearing moon, combined with the gravity signature of a deep ocean today (Hemingway et al. 2016; Beuthe et al. 2016; Zannoni et al. 2020) and the extensive extensional tectonics (Collins et al. 2010; Martin et al. 2016), suggest a long-lived ocean that has partially frozen over Dione’s surface history. Although models can produce past oceans within Dione, a contemporary ocean would require additional heat sources than have been incorporated into existing interior evolution models (Castillo-Rogez et al. 2018; Neveu and Rhoden 2019). Studies of fractures that would arise from ocean freezing may provide some constraints on the extent of freezing and/or the minimum thickness of Dione’s ice shell throughout its recorded history.

Finally, Dione’s surface displays polygonal, elliptical, and circular craters, including many large craters and many relaxed craters (Beddingfield et al. 2016; White et al. 2017; Ferguson et al. 2020, 2022b). These craters need both time and sufficient source populations to accumulate. As described in Sect. 3.3, Dione’s elliptical craters have the same geographical signatures as those on Tethys (Ferguson et al. 2022b), suggesting a common origin or process for their formation.

### Rhea

#### Overview of Surface Geology

**Fig. 11:**
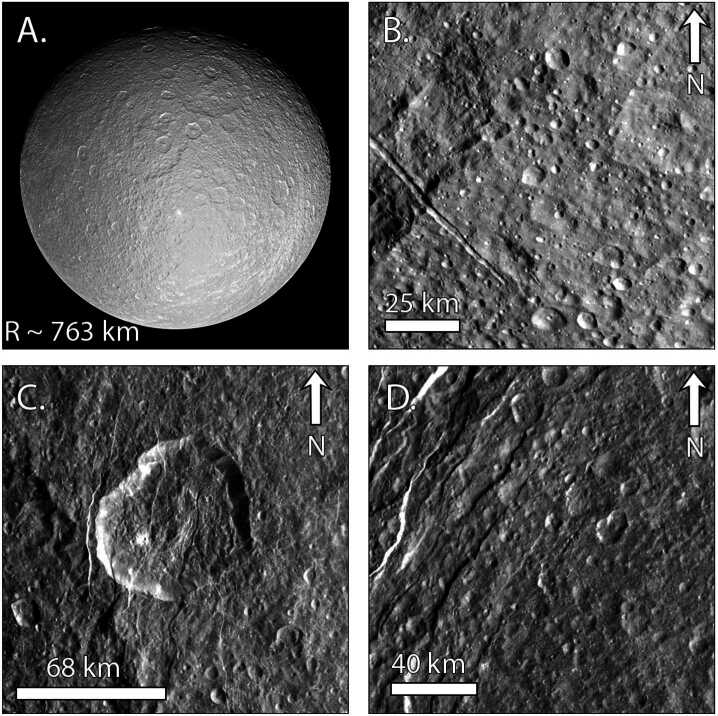
A) Full disk image of Rhea with the Tirawa basin located near the center. Image PIA07763. B) Cratered terrain with a linear feature. Image N1567132880_1. C) Obatala crater (62.5 km) overprinted by tectonics. Image N1637518901_1. D) Tectonic features alongside cratered terrain. Image N1637519610_1

Rhea’s surface (Fig. 11) is heavily cratered (e.g., Kirchoff and Schenk 2010; Schenk et al. 2020) and displays large, degraded basins such as Mamaldi (D = 480 km) and Powehiwehi (D = 271 km). These basins are, themselves, heavily cratered, supporting the idea that Rhea’s surface is much older than, for example, the cratered terrains of Enceladus (Kirchoff and Schenk 2009). There is some debate in the literature as to whether Rhea’s smaller craters record the same impactor population as the other mid-sized moons (e.g., Kirchoff and Schenk 2010 versus Bell 2020) and which craters are most likely to be heliocentric in origin (e.g., Kirchoff and Schenk 2010 versus Hirata 2016). Rhea has a dearth of large (D > 100 km) craters, as compared with Iapetus, leading to the suggestion that Rhea had such high heat flows early in its history that many early craters were relaxed beyond recognition (White et al. 2013).

As summarized by Schenk et al. (2018), tectonic features on Rhea include long (>1000 km), north-south trending canyons and a series of ridges and troughs. Many of these features are thought to be extensional, suggesting a formation mechanism related to a volume increase within the moon, although an older period of global contraction has also been suggested (e.g., Moore et al. 1985). Rhea’s heavily cratered surface obscures some older tectonic features, making it challenging to discern their origins (Schenk et al. 2018 and references therein). However, there are also several craters crosscut by more recent tectonic features, indicating different epochs of tectonic activity (Beddingfield et al. 2015; Aponte-Hernández et al. 2021).

Craters on Rhea with diameters greater than 100 km appear to all record similar amounts of crater relaxation, which require 10s of mW/m^2^ of heat (White et al. 2013). Regardless of the magnitude of the heat flow, White et al. (2013) find that the majority of resulting relaxation takes place within ∼10 Myr, with little additional change in crater depth even if the crater is heated for 1 Gyr. This result suggests that even a relatively short-lived pulse of enhanced heat flow could substantially relax craters on Rhea, although craters could have been exposed to high heat flows for much longer than 10 Myr without leaving a clear signature. Shallow slopes of normal faults on Rhea are also suggestive of viscous relaxation, implying elevated heat flows (Beddingfield et al. 2015), and Rhea’s global shape also indicates higher heat flows than expected (Nimmo et al. 2010).

#### Interior Structure and Evolution

Even with *Cassini* measurements, the structure of Rhea’s interior is still somewhat ambiguous. Rhea’ density is about 1.237 g/cm^3^, lower than Enceladus and Dione. The density corresponds to an ice fraction of 65 wt.% or 86 vol.%, assuming no porosity (Castillo-Rogez et al. 2018). The single initial gravity pass at Rhea led to some controversy as to whether it supported or excluded hydrostatic equilibrium (Anderson and Schubert, 2007; Iess et al. 2007; Mackenzie et al. 2008; Anderson and Schubert 2010), in part because the motion of the spacecraft must be very precisely characterized in order to isolate the gravitational effects of the single coefficients J_2_ and C_22_ of the moon. However, with a second flyby, there was sufficient data to determine that Rhea’s gravity field is significantly non-hydrostatic (Tortora et al. 2016), with J_2_/C_22_ of 3.91 ± 0.1. Hence, the structure of Rhea’s interior cannot be directly constrained. Combining the gravity data, plausible interior structures, and constraints on Rhea’s shape (Thomas et al. 2007; Thomas 2010; Nimmo et al. 2011), Tortora et al. (2016) showed that both differentiated and undifferentiated interiors are allowed within the current data set. Overall, they favor a model in which Rhea is differentiated with a low density, modestly non-hydrostatic core - perhaps similar to what has been suggested for Mimas (Tajeddine et al. 2014; Caudal 2017; Noyelles 2017).

Thermal-orbital evolution models have shown that a primordial Rhea, formed in the CPD, could have differentiated and maintained a short-lived ocean early in its history (Hussmann et al. 2006; Kamata and Nimmo 2015; Neveu and Rhoden 2019). The thermal evolution of a ring-born Rhea has not yet been explored as that scenario requires a Q of Saturn that can enable Rhea to migrate out to its current orbital distance within the lifetime of the Solar System. As our understanding of Q evolves (e.g., Lainey et al. 2020), there may be additional pathways for Rhea’s formation via Saturn’s rings. In that case, Rhea may have formed with a rocky interior and ice exterior without experiencing global melting (e.g., Charnoz et al. 2011; Salmon and Canup 2017).

As with Tethys and Dione, Rhea’s geology records higher heat flows than thermal models would have suggested (Passey 1983; Schenk 1989; Moore et al. 2004; Nimmo et al. 2010; White et al. 2013; Aponte-Hernández et al. 2021). The source of Rhea’s internal heating remains somewhat mysterious. Radiogenic heating alone is insufficient to explain the high heat flows recorded by craters (White et al. 2013; Kamata and Nimmo 2015). Rhea’s potential for tidal heating is limited due to its large distance from Saturn and low eccentricity, and Rhea is not currently in any mean motion resonances. If Rhea is differentiated, its core is likely non-hydrostatic (e.g., Tortora et al. 2016) and low density (∼2.4 g/cm^3^), perhaps similar to the “fluffy” core proposed for Enceladus (e.g., Roberts 2015). However, dissipation within the core would still require some kind of tidal forcing, which is again challenging due to the moon’s distance from Saturn.

In contrast to geologic inferences that Rhea is old, its orbital inclination is challenging to explain if Rhea is primordial (Ćuk et al. 2016), particularly given its current rapid orbital migration rate (Lainey et al. 2020). Passing through the so-called evection resonance, which is located within Rhea’s current orbital distance, should have pumped the moon’s inclination beyond what is currently observed, with no clear mechanism to subsequently damp it (Ćuk et al. 2016). The timing of Rhea’s passage through the resonance (100s of Myr ago versus billions of years ago) depends sensitively on how long Rhea’s orbital migration has been as rapid as observed today (e.g., Ćuk et al. 2016; Teodoro et al. 2023).

To address the issue of Rhea’s low inclination, Teodoro et al. (2023) suggested that a precursor moon evolved through the evection resonance, which resulted in its eccentricity and inclination being pumped and leading to a disruptive collision with a Dione-sized moon. Present-day Rhea then assembled from the collisional debris, with a low eccentricity and inclination, outside the evection resonance. Teodoro et al. (2023) assumed the event occurred very recently, such that the debris from the collision could supply material to Saturn’s rings. A challenge with this model, and any model of very recent formation or reassembly of Rhea, is how to produce the geologic history preserved in its surface, which implies multiple tectonic episodes, a significant crater population, and sufficiently high heat flows to relax craters and other features. Alternatively, if Rhea (or its precursor moon) passed through the evection resonance earlier in Saturn system history, and then disrupted and reassembled, there would be more time for it to accumulate its geologic record.

#### Summary of Constraints

Models of Rhea’s formation and evolution need to account for the high heat flows recorded by Rhea’s craters (White et al. 2013), shallow faults (Beddingfield et al. 2015), and long-wavelength topography (Kamata and Nimmo 2015). As with Tethys and Dione, the presence of extensional tectonics suggests a volume increase, which could be attributed to ocean freezing, although global contraction has not been ruled out either (see discussion in Schenk et al. 2018). In addition, the heat source to generate an ocean, and the likely timing of its existence, is not well constrained. There appear to be multiple epochs of tectonic activity on Rhea (e.g., Beddingfield et al. 2015; Aponte-Hernández et al. 2021), which could indicate long-term cooling, additional drivers of tectonic activity, or variability in heat flow, internal structure, and stress related to a complex orbital history. Rhea’s geologic record may provide additional constraints on its evolution, but more work is needed to analyze its surface features and their implications. Furthermore, there is a challenge in reconciling the relatively low inclination of Rhea with its heavily cratered, and presumed ancient, surface (e.g., Ćuk et al. 2016; Teodoro et al. 2023).

Finally, characterizing the population of elliptical craters on Rhea, similar to studies conducted for Mimas, Tethys, and Dione (Ferguson et al. 2022b, 2024), may help constrain the characteristics of planetocentric debris, which could help reduce uncertainty in crater-based ages. The lack of co-orbital moons at Rhea also provides a means of testing the role of co-orbital debris in cratering the mid-sized moons. In addition, an examination of polygonal craters may help identify older tectonics, as has been determined at Dione (Beddingfield et al. 2016).

### Summary of Geologic Constraints Across All Five Mid-Sized Moons

The interior structures of Mimas, Enceladus, and Dione are layered and have possessed internal heating sufficient to generate oceans that likely persist to the present day. At Mimas, observations of its librations and precession can only be explained by a differentiated interior, are most consistent with a subsurface ocean, and also support an elongated core (Tajeddine et al. 2014; Caudal 2019; Noyelles et al. 2019; Lainey et al. 2024). At Enceladus, there is definitive evidence of a global ocean and a low-density core (e.g., Thomas et al. 2016; Hemingway et al. 2018 and reference therein; Hemingway and Mittal 2019; Park et al. 2024), which has been interpreted as a porous core subject to hydrothermal circulation (e.g., Roberts 2015; Choblet et al. 2017; Rovira-Navarro et al. 2022). At Dione, where libration measurements are not available, interpretations of gravity and shape data suggest a deep ocean under a thick ice shell (Hemingway et al. 2016; Beuthe et al. 2016; Zannoni et al. 2020). Hence, models for the formation of Mimas, Enceladus, and Dione, need to account for their layered interiors and provide a pathway to develop their present-day oceans.

The data for Rhea is suggestive of a differentiated moon with some core topography, although a largely undifferentiated interior is also possible (Tortora et al. 2016). Unfortunately, the interior structure of Tethys is not well-constrained by direct measurements (Thomas 2010; Castillo-Rogez et al. 2018). However, as discussed below, there are inferences from geologic features on both Tethys and Rhea that suggest they once possessed oceans, implying compositional layering within their interiors.

There is evidence of either sustained or recurring epochs of tectonic activity on Enceladus (e.g., Patthoff and Kattenhorn 2011; Crow-Willard and Pappalardo 2015), Dione (e.g., Collins et al. 2010; Hammond et al. 2013; Beddingfield et al. 2016; Martin et al. 2016), and Rhea (e.g., Beddingfield et al. 2015; Aponte-Hernández et al. 2021). Diurnal tidal stresses have been linked to the formation of young fractures on Enceladus (e.g., Rhoden et al. 2020), while non-synchronous rotation (NSR) stresses have been suggested to explain older fracture sets on Enceladus (Patthoff and Kattenhorn 2011) and features on Dione (Collins et al. 2010; Martin et al. 2016). NSR would require a subsurface ocean to decouple the ice shell from the rocky interior (e.g., Helfenstein and Parmentier 1985). The extent to which diurnal tidal stress or stress from NSR has produced tectonic activity on Tethys or Rhea is not well-known.

Freezing of subsurface oceans has been invoked to facilitate the formation of extensional tectonic features observed on Enceladus, Tethys, Dione, and Rhea (e.g., Moore et al. 1985, 2004; Collins et al. 2010; Hemingway et al. 2020). Hence, these features indicate a heat source capable of generating an ocean, particularly if tidal stresses are involved in their formation, and subsequent reduction of the total heat budget in order to transition to cooling and freezing. Tectonic activity is minimal at Mimas, which is consistent with an emerging ocean or a frozen interior (Rhoden et al. 2017, 2024). Formation models for these moons must provide sufficient time, internal heating, and stress to drive thermal evolution of their interiors and the associated tectonic activity preserved at the surface.

Enceladus, Tethys, Dione, and Rhea all display geologic evidence of high heat flows (Spencer et al. 2006; Chen and Nimmo 2008; White et al. 2013; Hammond et al. 2013; White et al. 2017; Beddingfield et al. 2023; Gyalay and Nimmo 2023). There are comparatively fewer constraints on Mimas’ surface heat flow. The ∼10 craters so far examined show no clear signature of relaxation (Schenk 1989; White and Schenk 2011; Schenk et al. 2018), but Mimas’ low gravity leads to less topographic change for a given heat flow than other mid-sized moons, making Mimas’ craters less diagnostic than craters elsewhere (Rhoden et al. 2024; cf., Bland et al. 2012 for Enceladus).

Achieving high heat flows from tidal dissipation within these moons implies the presence of subsurface oceans, warm ice that responds to tides, and/or highly dissipative cores. In addition, past epochs of higher eccentricity (or obliquity, e.g., Chen et al. 2014) may be required in order to generate high enough tidal dissipation to match observations. This is particularly true for Tethys, which currently has negligible eccentricity. Orbital evolution models have shown that eccentricity-pumping resonance crossings can be produced under a wide variety of conditions, including high, low, or variable Q of Saturn and in cases assuming that the moons formed contemporaneously with Saturn or were spawned from the rings within the last billion years (e.g., Ćuk et al. 2016; Neveu and Rhoden 2019; Nakajima et al. 2019; Tian and Nimmo 2020; Nakajima et al. 2019; Ćuk and El Moutamid 2022).

Dissipation within Enceladus appears to be dominated by tidal heating in its core (Roberts 2015; Choblet et al. 2017; Rovira-Navarro et al. 2022). If the other mid-sized moons also have dissipative cores, it could affect their heat budgets, perhaps providing a source for the heat that relaxed craters on Tethys, Dione, and Rhea (White et al. 2013, 2017). However, the orbits of the moons must also be conducive to tidal forcing. The enhanced core dissipation comes from the assumption that the rocky core is highly porous, and that the pores are filled with ice (Roberts 2015) or liquid water that flows through the core (Rovira-Navarro et al. 2022). Preserving ice and/or porosity in the core may be a discriminator in testing models of the formation and thermal evolutions of the moons. Similarly, the cores of Mimas and Rhea are unlikely to be hydrostatic (Tajeddine et al. 2014; Caudal 2017; Tortora et al. 2016), which may help constrain their formation.

All of the mid-sized moons record extensive bombardment histories - even Enceladus, outside of the south polar terrain (e.g., Kirchoff and Schenk 2009, 2010). The crater SFDs indicate that planetocentric material played an important role in cratering Mimas, Enceladus, Tethys, and Dione, although the crater populations vary even across these moons (Kirchoff et al. 2018; Bell 2020; see also, Sect. 3). Although some studies suggest that all five mid-sized moons have been subjected to the same population of impactors (e.g., Bell 2020), differences in Rhea’s crater population have been interpreted as a clearer signature of heliocentric impactors relative to the other moons (e.g., Kirchoff and Schenk 2010; Hirata 2016).

The elliptical crater populations on Mimas, Tethys, and Dione display some key similarities, such as clustering of east-west oriented craters within 30° of the equator of each moon (Ferguson et al. 2022b, 2024). Given the present-day orbital inclinations of the moons, the similar location and extent of the elliptical craters suggests either individual impactor populations with similar dynamics or a common impactor population that predated the increased inclinations of the moons (e.g., Ferguson et al. 2022b). The observed elliptical crater populations provide constraints on the distribution of planetocentric material within the Saturn system.

In addition to the geophysical properties of the moons, their relationships with other components of the Saturn system may provide important constraints. Tethys and Dione each have co-orbital moons within their Lagrange points. Analyses of stability regions at Saturn suggest that Rhea, if it ever had co-orbitals, would have lost them upon crossing the evection resonance (Giuppone et al. 2018), while overlapping resonances may reduce stability of Lagrange points at Mimas and Enceladus (Christou et al. 2007). Hence, it is possible that more of the mid-sized moons had co-orbitals in the past. Although it may be tempting to dismiss these small moons, accounting for their existence can provide insight as to the formation and evolution of the mid-sized moons. Similarly, the on-going evolution of the Cassini Division may constrain the recent orbital changes at Mimas (Baillié et al. 2019; Noyelles et al. 2019), providing a potential pathway to form a young ocean within the moon (Lainey et al. 2024; Rhoden et al. 2024). And finally, characterizing the nature of Saturn’s interior and how it has evolved through time would enable more robust models of Saturn’s Q, which controls the orbital expansion of the mid-sized moons, resonances passages, and the orbital properties that control tides. Narrowing down the possible thermal-orbital pathways for the moons can help pinpoint where additional mechanisms are needed to explain their geophysical characteristics.

## Synthesis and Conclusions

With the geologic constraints in mind (i.e., Sects. 3, 4, and 5), we now connect them – to the extent possible – to the proposed formation mechanisms for the mid-sized moons (Sect. 2). Specifically, we consider the mass gradient of the moons, their global shapes and interior structures, inferences as to their thermal histories, their craters, and clues as to the dynamical evolution of the Saturn system.

*Mass gradient*. Although accretion within Saturn’s CPD (Canup and Ward 2006, 2009; Mosqueira et al. 2010) or formation via mergers of larger planetesimals (Asphaug and Reufer 2013) cannot be ruled out, these models do not provide a clear pathway to explain the mass gradient of the moons. In contrast, ring-based formation of Saturn’s mid-sized moons can explain the mass, rather than density, variation of the moons with distance from Saturn (e.g., Crida and Charnoz 2012). If the moons formed from rings billions of years ago, they would either need to survive disruptive impacts or reassemble in a manner that preserves the mass gradient we currently observe. If the rings formed during a heavy bombardment period, such as by tidal disruption of a passing Centaur (e.g., Dones 1991; Hyodo and Charnoz 2017), the moons would emerge later in Solar System history and the likelihood of avoiding disruptive collisions with heliocentric impactors increases.

If any or all of the mid-sized moons emerged from the rings, their formation age is tied to the ring age. While the mass of the present-day rings suggests it has evolved over billions of years (Salmon et al. 2010), other observations imply an age on the order of 100 Myr (e.g., Zhang et al. 2017; Iess et al. 2019). If confirmed, such a young age for the rings would all but rule out direct assembly of the present-day moons from the present-day rings. In that case, we suggest that the Saturn system may have experienced cycles of ring formation, moon emergence, and ring replenishment via moon disruption, which could help explain the signature of ancient rings within the current ring mass (Salmon et al. 2010; see also Sect. 2).

*Global shape and interior structure*. Where *Cassini* data was sufficient to assess the interiors of the mid-sized moons, all of them show deviations from hydrostatic equilibrium in global shape, core shape, and/or gravity (e.g., Tajeddine et al. 2014; Thomas et al. 2016; Tortora et al. 2016; Zannoni et al. 2020), which may be an indication as to the conditions in which they formed. Moons that formed in the CPD and differentiated due to internal heating may produce a different interior structure than reassembly after mergers or ring-based formation models. For example, ring-based models may produce layered moons without invoking global internal melting and hydrothermal alteration (e.g., Neveu and Rhoden 2019), which may result in different shapes and interior structures than are typically assumed for differentiated moons (e.g., Sect. 4). The formation models also differ in the source of rock within the moons and how it would evolve into a core. Even the ring-based formation models have multiple approaches to introducing rocky material (e.g., Charnoz et al. 2011 versus Salmon and Canup 2010). Studies that track the thermal and physical evolution of the cores or/or global shapes of the moons across different moon formation models would be highly valuable. In addition, testing whether non-hydrostatic cores can be retained through disruptive collisions could help determine whether the moons have avoided such collisions.

*Inferred thermal histories*. There is evidence that Mimas, Enceladus, and Dione possess present-day oceans (Hemingway and Mittal 2019; Tajeddine et al. 2014; Thomas et al. 2016; Zannoni et al. 2020; Lainey et al. 2024). In addition, Enceladus, Tethys, Dione, and Rhea all display relaxed craters that indicate epochs of high heat flows (Bland et al. 2012; White et al. 2013, 2017). Freezing of ocean material has been suggested as a mechanism to drive tectonic activity on Enceladus (e.g., Hemingway et al. 2020; Rudolph et al. 2022), may have contributed to the formation of Ithaca Chasma on Tethys (Moore and Ahern 1983), and has been suggested as a source of extensional tectonics on Dione (Collins et al. 2010). At Rhea, there is no unambiguous evidence of a differentiated interior (e.g., Tortora et al. 2016), although its extensional tectonic features and highly relaxed craters have been linked to a past ocean, which would imply compositional layering in its interior (e.g., White et al. 2013).

If all of the mid-sized moons have/had oceans, it implies that the moons either underwent global differentiation or assembled layered (e.g., from rings). Oceans may form as part of the early interior evolution of a moon and/or during a later epoch of enhanced internal heating. Using combinations of accretionary, radiogenic, and tidal heating under different formation scenarios, thermal-orbital co-evolution models of all five moons can produce oceans at some point within the lifetimes of Enceladus, Tethys, Dione, and Rhea (Neveu and Rhoden 2019). Whether born from the CPD or Saturn’s rings, the simulations begin billions of years ago (e.g., Neveu and Rhoden 2019). Separate studies that focus only on the past ∼10 Myr of Mimas’ evolution have shown that a young ocean can be generated within Mimas by invoking a relatively recent increase in its eccentricity (Rhoden et al. 2024; Lainey et al. 2024). A critical open question is whether the development and, in some cases loss, of oceans can be reproduced in very young moons, particularly after disruptive collisions and reassembly.

The effectiveness of tidal heating to grow or preserve oceans and to relax topography depends on the responsiveness of the interior to deformation and the eccentricity, obliquity, and physical librations of the moon, which can be forced by mean motion resonances. In order to match the observed heat flows at Enceladus, some models incorporate a tidally dissipative core (e.g., Roberts 2015). We do not yet know whether other mid-sized moons possess dissipative cores or how porous cores may be formed and retained across different formation models. At Mimas, Tethys, and Dione, past epochs of higher eccentricity or obliquity that promote tidal heating have been suggested to account for the presence of an ocean and/or high heat flows (e.g., Mimas: Lainey et al. 2024; Rhoden et al. 2024; Tethys: Chen and Nimmo 2008; Dione: White et al. 2017). Hence, models for the formation of the mid-sized moons, and their subsequent thermal-orbital evolution, must provide opportunities for tidal dissipation consistent with these observations and inferences.

Given the currently available models, we cannot yet tie the geologic records and thermal evolutions they imply directly to an initial formation model. With additional studies, the extent of geologic activity could be used to place constraints on the ages of the moons. For example, some histories may be ruled out by the resonance crossings required to match the heat flows recorded on the moons. Additional models are needed to connect dynamical evolution of the moons – accounting for things like the addition or loss of moons over time or different initial interior structures based on the proposed formation models – in order to fully utilize the geologic constraints. These models will also depend on, and may thus help to constrain, the historic Q of Saturn and its potential evolution through time.

*Craters*. Mimas, Tethys, Dione, and Rhea are heavily-cratered, as is Enceladus outside the south polar terrain (e.g., Kirchoff and Schenk 2009, 2010). These crater populations need time and source material to develop, which may be challenging if the moons are extremely young. However, the lack of craters at the largest sizes, as compared with Iapetus and model predictions for early heliocentric bombardment, suggest that the surfaces are not primordial either (Kirchoff and Schenk 2010; Bottke et al. 2024).

It may be difficult to preserve primordial mid-sized moons through early heavy bombardment (e.g., Movshovitz et al. 2015; Bottke et al. 2023, 2024), particularly if all of the moons formed contemporaneously in the CPD. Moons that emerged from the rings (e.g., Charnoz et al. 2011; Crida and Charnoz 2012; Salmon and Canup 2017) would have entered the system later than CPD moons, although most ring-based formation models still have the process begin billions of years ago. Whether that delay is sufficient to “save” the mid-sized moons from disruptive collisions by external impactors may depend on exactly when the rings formed and how many moons are assumed to emerge. Alternatively, one or more of the mid-sized moons may have reassembled from a disruptive collision due to early heliocentric bombardment, regardless of how it initially formed.

There is strong evidence of planetocentric debris in the Saturn system (e.g., Kirchoff et al. 2018), particularly as recorded in the distribution of elliptical craters (Ferguson et al. 2022b, 2024), for which collisions into or among existing moons seems the most likely source (e.g., Ćuk et al. 2016; Teodoro et al. 2023). An important consideration is the extent to which these collisions reset the surface geologic records of the moons, as well as their internal structures and eccentricities, and how much material is reaccreted versus lost to Saturn (e.g., Hyodo and Charnoz 2017, versus Teodoro et al. 2023). Additional modeling is needed to quantify the outcomes of hypothesized collisions for comparison with the moons.

*Dynamical evolution*. Looking at the Saturn system more broadly, there appears to be evidence of relatively recent dynamical changes. Dynamic destabilization of one or more moons, leading to collisions destructive enough to generate a planetocentric population of impactors, is the current prevailing hypothesis for the source of the material that cratered the mid-sized moons (e.g., Wisdom et al. 2022; Teodoro et al. 2023). However, the timing and longevity of planetocentric bombardment is somewhat uncertain. Elliptical craters, which are thought to be planetocentric in origin, seem to be less prevalent in the youngest regions of Tethys and Dione, as compared with more heavily cratered regions (Ferguson et al. 2022b), perhaps because the impactor population became depleted within the time frame recorded on their surfaces. Furthermore, the similarities in elliptical crater populations at Tethys and Dione suggest the craters predate inclination pumping of Tethys, although other options are plausible (Ferguson et al. 2022b). Therefore, it seems likely that whatever generated planetocentric material at Saturn predated at least some of the geological and dynamical evolution of the moons.

The fact that Rhea currently orbits outside the evection resonance, while having a relatively low inclination, suggests either dynamically-driven destruction and reassembly to produce the present-day Rhea or a complex series of resonances that can accommodate the dynamical effects of evection (e.g., Ćuk et al. 2016; Teodoro et al. 2023). In either case, Rhea’s heavily cratered surface is more consistent with an ancient surface than a young one (e.g., Kirchoff and Schenk 2010). Identifying pathways that reproduce Rhea’s present-day low inclination may provide useful constraints on the overall evolution of the mid-sized moons.

If Mimas’ ocean is confirmed by additional measurements, it implies recent (∼10 Myr) eccentricity pumping of Mimas (Lainey et al. 2024; Rhoden et al. 2024). Furthermore, Mimas’ recent orbital evolution may be related to the on-going evolution of the Cassini Division (e.g., Baillié et al. 2019), and Mimas is in an inclination-type resonance with Tethys. Pinning down the timing and conditions over which Saturn’s rings, Mimas, and Tethys co-evolved would be useful for understanding the evolution of the moons and may also help constrain the lifetime of Saturn’s present ring system. Additional measurements or experiments that can date the rings would also be helpful in constraining formation models of the rings and mid-sized moons.

*Conclusions*. The *Cassini* mission enabled characterization of the surfaces of the mid-sized moons and, to varying extent, their interior structures and thermal histories. As more sophisticated models for the initial formation of the mid-sized moons, and subsequent collisions and reassembly, are developed, we can better test and identify the scenarios most consistent with these constraints. Of particular value would be identifying the starting locations of the mid-sized moons, such that their past resonance passages and overall tidal dissipation can be constrained. Better estimates of the present orbital migration rates of the moons, as well as information about Saturn’s interior that governs its dissipation over time, can aid in this endeavor. While the ages of the mid-sized moons cannot yet be firmly determined by their cratering records or inferred thermal evolution, neither extreme in age (primordial or ∼100 Myr) appears fully consistent with the observations. Hence, we consider middle-aged moons to be most plausible and encourage continued investigation into formation and/or reassembly models that would allow retention of geologic features over billion year timescales.
